# Zoledronic acid inhibits the growth of cancer stem cell derived from cervical cancer cell by attenuating their stemness phenotype and inducing apoptosis and cell cycle arrest through the Erk1/2 and Akt pathways

**DOI:** 10.1186/s13046-019-1109-z

**Published:** 2019-02-21

**Authors:** Li Wang, Yanyang Liu, Yueling Zhou, Jiantao Wang, Li Tu, Zhen Sun, Xiujie Wang, Feng Luo

**Affiliations:** 1 Lung Cancer Center, Laboratory of Lung Cancer, West China Hospital of Sichuan University, Chengdu, 610041 Sichuan China; 2 Department of Medical Oncology, Cancer Center and State Key Laboratory of Biotherapy, West China Hospital of Sichuan University, Chengdu, 610041 Sichuan China; 3 Laboratory of Experimental Oncology, State Key Laboratory of Biotherapy, West China Hospital of Sichuan University, Chengdu, 610041 Sichuan China

**Keywords:** Zoledronic acid, Cancer stem cell, Cervical cancer, Erk1/2, PI3K/Akt

## Abstract

**Background:**

Zoledronic acid is the most potent osteoclast inhibitor and is widely used for advanced cancer patients with bone metastasis, but its role on cancer stem cells (CSCs) remains unclear. In the present study, we aimed to identify the stemness phenotypic characteristics of CSCs derived from cervical cancer cells and explore the anti-cancer efficiency of zoledronic acid on these cells, as well as the possible molecular mechanisms.

**Methods:**

Stemness phenotypic identification of cervical cancer cells derived CSCs was performed via sphere formation efficiency (SFE), tumorigenesis, immunofluorescence staining, Transwell assay, and western blot. Anti-cancer efficiency of zoledronic acid on these cells (including proliferation, stemness phenotype, apoptosis, and cell cycle) was carried out through MTT assay, SFE, transwell, DAPI staining, flow cytometry, immunofluorescence, TUNEL staining, and western blot, both in vitro and in vivo.

**Results:**

Enhanced self-renewal ability, including SFE and tumorigenesis, was verified in cervical cancer cells derived CSCs compared to parental cervical cancer cells. Specifically, the expression of ALDH1, Sox2, CD49f, Nanog, and Oct4 was significantly up-regulated in cervical cancer cells derived CSCs. Furthermore, enhanced migratory ability was observed in these cells along with up-regulated N-cadherin and Vimentin and down-regulated E-cadherin. Zoledronic acid inhibited cervical cancer cells derived CSCs proliferation in vitro and in vivo. The stemness phenotype of these CSCs including tumor sphere formation, migration, as well as the expression of the aforementioned associated markers was also suppressed. In addition, zoledronic acid significantly induced apoptosis and cell cycle arrest of cervical cancer cells derived CSCs in a dose-dependent manner. Mechanistically, the expression of phosphorylated Erk1/2 and Akt was significantly increased in cervical cancer cells derived CSCs compared to parental cervical cancer cells. Zoledronic acid inhibited phosphorylated Erk1/2 and Akt in cervical cancer cells derived CSCs. IGF-1, a potent stimulator for Erk1/2 and PI3K/Akt, attenuated the aforementioned anti-cancer effect of zoledronic acid.

**Conclusions:**

Zoledronic acid inhibited the growth of cervical cancer cells derived CSCs through attenuating their stemness phenotype, inducing apoptosis, and arresting cell cycle. The suppression of phosphorylated Erk1/2 and Akt was involved in this process.

**Electronic supplementary material:**

The online version of this article (10.1186/s13046-019-1109-z) contains supplementary material, which is available to authorized users.

## Background

Cervical cancer is the fourth most common cancer in women globally and the second most common in developing countries [1]. The number of patients diagnosed with cervical cancer has increased in recent years due to improvements of healthcare infrastructures and screening programs [2]. The current therapeutic strategies include surgical resection, conventional chemotherapy, radiotherapy, and human papillomavirus (HPV) vaccines [3, 4] and are effective against cervical cancer, but their curative effects are limited. Most patients with cervical cancer ultimately have cancer recurrence and metastasis, with subsequent mortality.

Cancer stem cells (CSCs) exist as a small subpopulation of cells within many types of tumors or cancer cell lines including lung cancer, breast cancer, prostate cancer, endometrial cancer, and leukemia [5–7]. These heterogeneous cell populations possess stem cell properties such as enhanced self-renewal ability, chemo/radio-resistance, epithelial mesenchymal transition (EMT), multi-differentiation potential, and expression of specific stemness associated markers (ALDH1, Sox2, Oct4 and Nanog, CD133, etc.), and are considered to be closely involved with cancer prognosis [8–10]. At present, emerging evidence indicates that CSCs in tumors or hematological neoplasms are responsible for cancer recurrence and metastasis, irrespective of anti-cancer therapies. Fortunately, compared to cancer cells, the specific markers and signal transduction networks of CSCs might offer opportunities to eliminate or induce differentiation of CSCs, ultimately improving prognosis [11, 12]. Therefore, developing new targeted drugs or exploring new mechanisms of existing drugs targeting specific markers or signal transduction networks of CSCs is a promising anti-cancer therapeutic strategy.

Zoledronic acid is an imidazole-containing bisphosphonate and is widely used in clinic due to its direct therapeutic effect on osteoclasts and the prevention of skeletal-related events in cancer patients with bone metastasis [13]. Recent reports confirmed that in addition to the direct functional roles on cancer cells (including apoptosis induction, cell cycle arrest, and autophagy), zoledronic acid modulates the tumor microenvironment through regulating angiogenesis and immunity [14–16], indicating that zoledronic acid might be a multi-target drug acting on several signaling pathways. The efficacy of zoledronic acid has been verified in breast cancer [17], lung cancer [18, 19], pancreatic cancer [20, 21], colon cancer [22], and glioma [23, 24]. Nevertheless, whether zoledronic acid has possible anti-cancer functional roles on CSCs remains unknown. In the present study, we aimed to identify the stemness phenotypic characteristics of cervical cancer cells derived CSCs and investigate the potential anti-cancer efficiency of zoledronic acid (including stemness phenotypic attenuation, apoptosis induction, and cell cycle arrest) on these cells. In addition, we explored the possible mechanisms and signaling pathways.

## Methods

### Cell culture

The human cervical cancer cell lines HeLa, SiHa, and CaSki were obtained from the Shanghai Cell Biology Institute of the Chinese Academy of Sciences (Shanghai, China). These cells were maintained in Dulbecco’s modified Eagle’s medium (DMEM) supplemented with 10% fetal bovine serum (FBS), penicillin (100 U/ml), and streptomycin (100 μg/ml) at 37 °C in the presence of 5% CO_2._

The HeLa cells derived CSCs have been previously established [8] and cryopreserved in our laboratory. We resuscitated and cultured these cells using a non-adhesive culture system previously described [8]. Briefly, cells were seeded in 10-cm culture dishes pre-coated with 0.5% agarose at a density of 1 × 10^6^ cells and cultured with DMEM medium containing 10% FBS, penicillin (100 U/ml), and streptomycin (100 μg/ml) at 37 °C in the presence of 5% CO_2_. The medium was changed every other day until tumor sphere formation within 5–7 days. Tumor spheres were dissociated with Accutase (Gibco/BRL Invitrogen, Shanghai, China) to generate single cells. In addition, SiHa and CaSki cells derived CSCs were enriched using the non-adhesive culture system, as mentioned above.

### Stemness characterization assays in cervical cancer cells derived CSCs

#### Tumor sphere formation assay

To assess tumor sphere formation efficiency (SFE), single-cell suspensions derived from cervical cancer cells derived CSCs or parental cervical cancer cells were plated on 24-well plates (pre-coated with 0.5% agarose) at a density of 50 or 100 cells per well. After 12 days of culture, the sphere number (> 50 cells) in each well was counted under the microscope. SFE was calculated as the number of spheres formed divided by the initial number of single cells plated, and was expressed as a percentage.

#### Immunofluorescence for cells

Parental HeLa cells or HeLa cells derived CSCs were fixed with 4% paraformaldehyde for 10 min, permeated using 0.1% Triton X-100, and blocked with 5% BSA in phosphate buffered saline (PBS) for 30 min at room temperature. The cells were incubated with primary rabbit antibodies against ALDH1, Sox2, Oct4, Nanog, and CD49f (1:300, Beijing Biosynthesis Biotechnology Co., LTD, Beijing, China) at 4 °C overnight. After washing in PBS, the cells were incubated with goat anti-rabbit secondary antibodies conjugated with FITC or RBITC (Beijing Biosynthesis Biotechnology Co., LTD, Beijing, China) diluted 1:500 in PBS for 1 h at room temperature. DAPI (Sigma-Aldrich, USA) was used for nuclear staining. Images were obtained using a Leica DM1400B inverted fluorescence microscope with a DFC340FX camera.

#### Transwell assay

Cervical cancer cells derived CSCs or parental cervical cancer cells were resuspended in serum-free DMEM at a density of 3 × 10^5^ cells/mL in Transwell inserts (8-μm membrane, Corning), with DMEM containing 15% FBS in the bottom of a 24-well plate. The Transwell membranes were fixed in 4% paraformaldehyde after 24 h of culture at 37 °C with 5% CO_2_ and stained with crystal violet. The non-migrated cells on top of the Transwell membrane were removed with a cotton swab. The migrated cells on the bottom of the Transwell membrane were visualized and counted from five random fields under a inverted microscope equipped with a photocamera (Leica, Japan).

#### Western blot

Cells or tumor tissues were collected and lysed using RIPA lysis buffer supplemented with a protease and phosphatase inhibitor cocktail (Beyotime Biotechnology). Cell lysates in 1× loading buffer were resolved by 12% SDS-PAGE and transferred on polyvinylidene fluoride membranes (Milipore, Bedford, MA, USA). After blocking with 5% skimmed milk for 1 h, the membranes were incubated with primary rabbit antibodies against β-actin, Sox2, Oct4, ALDH1, Nanog, CD49f (1:300, Beijing Biosynthesis Biotechnology Co., LTD, Beijing, China), Vimentin, E-cadherin, and N-cadherin (1:1000, Proteintech Group, Chicago, USA) overnight at 4 °C. The primary antibodies were detected with peroxidase-conjugated goat anti-rabbit IgG (H + L) secondary antibody (Zhongshan Goldenbridge Biotechnology Co., Ltd., Beijing, China). Positive signals were detected using the BeyoECL Plus kit (Beyotime Institute of Biotechnology, Shanghai, China) and a western blot analysis system (Universal Hood II, Bio-Rad, USA).

#### Tumorigenicity assay in immunodeficient mice

To evaluate the tumorigenicity of parental HeLa cells and HeLa cells derived CSCs, 10^3^, 10^4^, 10^5^, and 10^6^ of HeLa cells or 10^2^, 10^3^, 10^4^, and 10^5^ of HeLa cells derived CSCs were suspended in 100 μL PBS and subcutaneously inoculated into female BALB/c nude mice (5-week-old, 20 ± 2 g, Hua Fu Kang Biotechnology Co. Ltd., Beijing, China, *n* = 4/group). Four weeks after inoculation, the mice were sacrificed and the tumor tissues were collected. All animal experiments were approved and carried out in accordance with the guidelines of Institutional Animal Care and Use Committee of Sichuan University.

### Anti-cancer efficiency of zoledronic acid on cervical cancer cells derived CSCs

#### Cytotoxic assay

Cervical cancer cells derived CSCs or parental cervical cancer cells were seeded in 100 μL of medium/well with 2 × 10^3^ cells/well in 96-well plates (pre-coated with or without 0.5% agarose). After incubation with different concentrations of zoledronic acid (Basel, Switzerland) (5, 10, 20, 40, and 80 μM) for 1, 2, 3, 4, and 5 days, 10 μL of MTT (pH 4.7) were added to each well and incubated for 4 h. Then, 100 μL of 10% SDS/0.01 N HCL were added to each well and incubated at 37 °C overnight to dissolve the formazan. Absorbance was measured at 570 nm. The effects of zoledronic acid on the viability of cervical cancer cells or cervical cancer cells derived CSCs were expressed as %cytoviability, using the following formula: %cytoviability = A_570_ of treated cells/A_570_ of control cells × 100%.

#### *Tumor sphere formation assa*y

The tumor sphere formation inhibition by zoledronic acid was determined by seeding 50 or 100 cervical cancer cells derived CSCs or parental cervical cancer cells per well in 24-well plates (pre-coated with 0.5% agarose) and incubated overnight. These cells were then treated with 10, 20, and 30 μM zoledronic acid, respectively. After 12 days of incubation, the sphere number (> 50 cells) of each well was counted and captured using microscope equipped with a photocamera. SFE was calculated as mentioned above.

#### Transwell assay

Cervical cancer cells derived CSCs or parental cervical cancer cells were pretreated with 10, 20, and 30 μM zoledronic acid respectively for 72 h and then harvested for Transwell assay, as above.

#### DAPI staining for apoptotic cells

Cervical cancer cells derived CSCs or parental cervical cancer cells were pre-treated with zoledronic acid (10, 20, and 30 μM, respectively) for 72 h. The cells were harvested, fixed in neutral formalin, treated with 0.1% Triton X-100 in PBS for 15 min at room temperature, and stained with 50 μL of DAPI (4 mg/mL, Sigma-Aldrich) for 30 min at room temperature. After washing with PBS, the samples were captured and counted from five random fields under a fluorescence microscope (Leica, Japan).

#### Annexin V-FITC apoptosis assay

Parental HeLa cells or HeLa cells derived CSCs pre-treated with zoledronic acid (10, 20, and 30 μM, respectively) for 72 h were harvested and washed twice with PBS. These cells were resuspended in 500 μL of binding buffer containing 5 μL of FITC-labeled Annexin-V and 5 μL of PI solution (BD Company). The tubes were kept on ice for 15 min and subjected to flow cytometry (Beckman Coulter) to assess the percentage of apoptotic cells.

#### Cell cycle

Cervical cancer cells derived CSCs or parental cervical cancer cells pre-treated with zoledronic acid (10, 20, and 30 μM, respectively) for 72 h were harvested and fixed in cold 70% ethanol at − 4 °C overnight. Subsequently, the cells were incubated with 50 ng/mL PI staining solution and 0.1 mg/mL RNase A in the dark for 30 min at room temperature. The DNA content of these cells was analyzed using a flow cytometer.

#### Xenograft

Five-week-old female BALB/c nude mice were purchased from Hua Fu Kang Biotechnology Co. Ltd. (Beijing, China). HeLa cells derived CSCs (1 × 10^5^) were suspended in 100 μL of PBS, subcutaneously inoculated in the mice, and allowed to grow for 7 days to reach a tumor volume of approximately 50–100 mm^3^. The mice were then randomly divided into four groups (*n* = 6/group). The treatment group animals were treated with zoledronic acid at concentrations of 20, 40, and 80 μg/kg, and the control animals were treated with an equal volume of PBS by intraperitoneal injection every day for 21 days. Tumor growth was monitored by measuring the tumor size every 3 days with a digital caliper. The tumor volume was calculated as V = 1/2 × (length×width^2^). The mice were sacrificed, and the tumor tissues were removed and weighted. All animal experiments were approved and carried out in accordance with the guidelines of the Institutional Animal Care and Use Committee of Sichuan University.

#### Immunofluorescence of tumor tissues

The tumor tissues were fixed in 4% paraformaldehyde and embedded in OCT compound (Tissue Tek) at 4 °C. Frozen section (4-μm thick) were plated onto matrigel-coated glass coverslips and permeabilized with 0.5% Triton X-100 for 10 min at room temperature, washed with PBS, and blocked in 5% BSA for 30 min before incubation with primary rabbit antibodies against ALDH1, Nanog, CD49f (1:100, Beijing Biosynthesis Biotechnology Co., Ltd., Beijing, China), and E-cadherin (1:500, Proteintech Group, Chicago, USA) overnight at 4 °C, followed by fluorescence-tagged secondary antibodies against rabbit IgG (1:500). Images were obtained using a fluorescence microscope (Leica, Japan).

For the TUNEL assay, the apoptotic cells in tumor tissues were detected using a One Step TUNEL apoptosis assay kit (Beyotime Institute of Biotechnology, Shanghai, China) according to the manufacturer’s protocol. The pretreatment procedures for the tissues were the same as for immunofluorescence, and the sections were labeled using the TdT reaction and incubated for 1 h at 37 °C. Then, Hocest33342 (Beyotime Biotechnology) was used for nuclear staining. The apoptotic cells were then visualized and counted from five randomly selected fields under a Leica inverted fluorescence microscope.

#### Western blot

After treatments, the cells or tumor tissues were collected and lysed using the RIPA lysis buffer supplemented with a protease and phosphatase inhibitor cocktail (Beyotime Biotechnology). The primary rabbit antibodies against β-actin, GAPDH, Bcl-2, Bax, Cleaved caspase-3 (1:300 Beijing Biosynthesis Biotechnology Co., LTD, Beijing, China), CyclinD1, CDK4, Erk1/2, pho-Erk1/2, Akt, pho-Akt, p38, pho-p38, PI3K (1:1000, Cell Signaling Technology, Inc. Danvers, USA), JNK, and pho-JNK (1:1000, Abcam, MA, USA) were used as above.

#### IGF-1 treatment

IGF-1 (R&D, Minneapolis, MN, USA) was used at a concentration of 200 ng/mL. Briefly, after HeLa cells derived CSCs were treated with or without zoledronic acid (30 μM) for 72 h, IGF-1 was added for another 24 h, and then the Transwell assay, DAPI staining, cell cycle, western blot, and SFE analyses were carried out, as above.

### Statistical analysis

Data were analyzed using Graphpad Version 6.0 (USA). Data are expressed as means ± standard deviation (mean ± SD). Significant differences among groups were analyzed using the Student’s *t*-test and one- or two-way ANOVA, as appropriate. *P* value < 0.05 was considered statistical significant.

## Results

### Cervical cancer cells derived CSCs exhibit stemness phenotypic characteristics

**Fig. 1:**
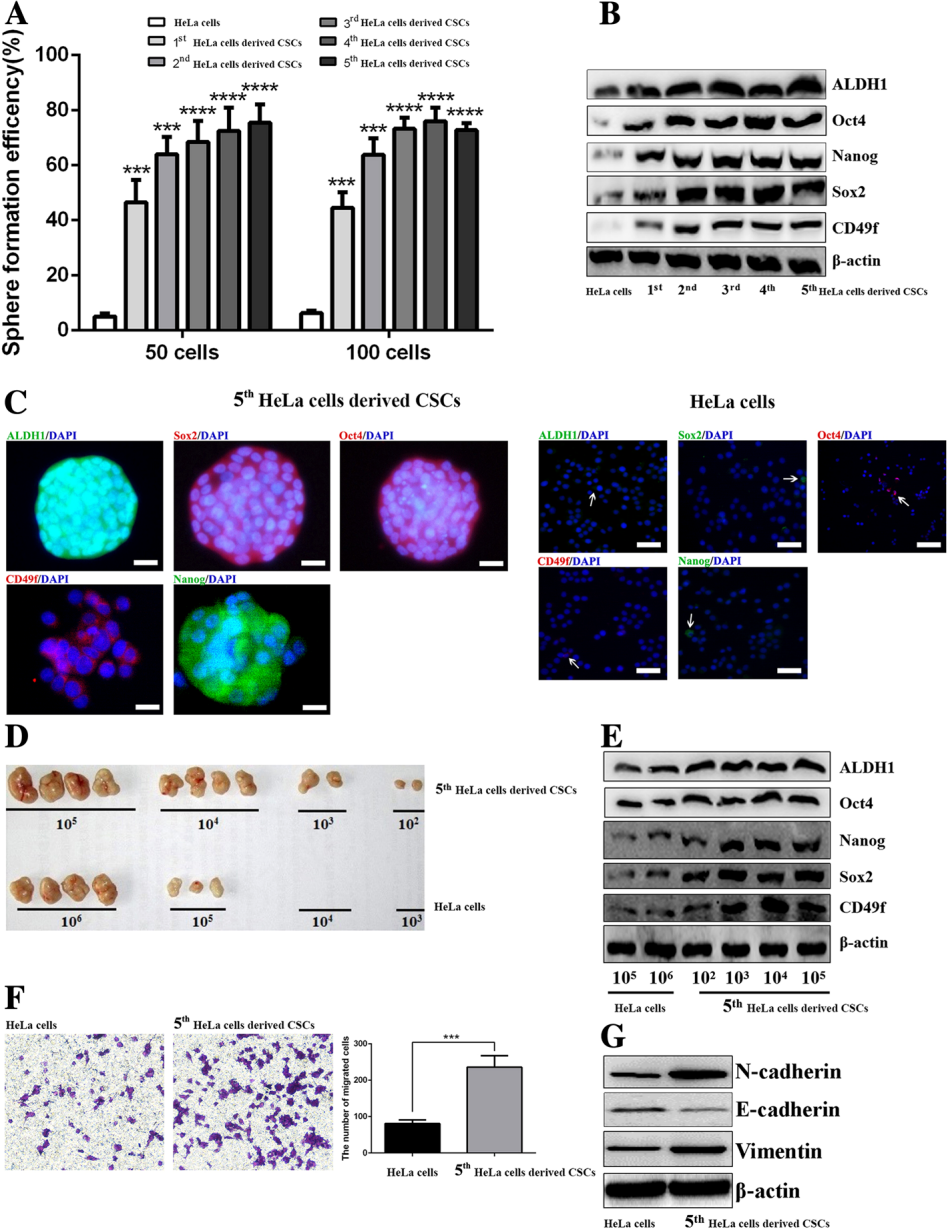
Resuscitated HeLa cells derived CSCs show stemness phenotypic characteristics**.** The graph shows the SFE of 1^st^ to 5^th^- passaged HeLa cells derived CSCs and parental HeLa cells **(a)**. Western blot analysis of ALDH1, Sox2, CD49f, Nanog, and Oct4 in 1^st^ to 5^th^-passage HeLa cells derived CSCs and parental HeLa cells **(b)**. Immunofluorescence staining of ALDH1, Sox2, CD49f, Nanog, and Oct4 in 5^th^-passage HeLa cells derived CSCs and parental HeLa cells, respectively; the white arrows point to positive cells **(c)**. Injection of different density of 5^th^-passage HeLa cells derived CSCs and parental HeLa cells generated xenografts in nude mice **(d).** Western blot analysis of ALDH1, Sox2, CD49f, Nanog, and Oct4 in tumor tissues derived from 5^th^-passage HeLa cells derived CSCs or HeLa cells bearing mice **(e)**. Transwell assay showing the migrated cells of 5^th^-passage HeLa cells derived CSCs and parental HeLa cells; the histogram shows the number of migrated cells; original magnification, × 400 **(f)**. Western blot analysis of E-cadherin, Vimentin, and N-cadherin in 5^th^-passage HeLa cells derived CSCs and parental HeLa cells **(g)**. * *P* < 0.05, ** *P* < 0.01, *** *P* < 0.001. Scale bars represent 50 μM or 10 μM in inset. Results are shown as mean values ± SD of independent experiments performed in triplicate

In order to verify the stable stemness phenotypic characteristics of HeLa cells derived CSCs cryopreserved in our laboratory, we resuscitated these CSCs and demonstrated their stemness phenotype through continuous passages. First, we detected the self-renewal ability in vitro by analyzing SFE. As shown in Fig. 1a, the SFE of 1^st^ to 5^th^ passage HeLa cells derived CSCs was obviously higher than in parental HeLa cells. Moreover, through western blot analysis, we demonstrated that the expression of ALDH1, CD49f, Sox2, Nanog, and Oct4 was higher in 1^st^ to 5^th^ passage HeLa cells derived CSCs compared to parental HeLa cells and tended to be stable in 5^th^-passage HeLa cells derived CSCs (Fig. 1b). Therefore, we chose the 5^th^-passage HeLa cells derived CSCs for further assessment of the stemness phenotypic characteristics. Using immunofluorescence, the fluorescence of ALDH1, CD49f, Sox2, Oct4, and Nanog in HeLa cells derived CSCs was obviously higher than in parental HeLa cells (Fig. 1c).

Tumorigenicity is considered as the gold standard for the identification of CSCs. The results revealed that 10^2^, 10^3^, 10^4^, and 10^5^ of HeLa cells derived CSCs generated tumors in 2/4, 2/4, 4/4, and 4/4 mice, respectively; while 10^3^, 10^4^, 10^5^, and 10^6^ of parental HeLa cells generated tumors in 0/4, 0/4, 3/4, and 4/4 mice. Further analysis of the stemness-associated markers in above tumor tissues showed that the expression levels of ALDH1, CD49f, Sox2, Nanog, and Oct4 were also higher in HeLa cells derived CSCs-derived tumor tissues compared to HeLa cells-derived tumor tissues (Fig. 1d-e). EMT is another trait of CSCs and indicates the acquirement of stemness phenotypes such as enhanced migration. In the present study, we verified that the 5^th^-passage HeLa cells derived CSCs were endowed with enhanced migration ability (Fig. 1f) along with obviously up-regulated expression of N-cadherin and Vimentin, and down-regulated E-cadherin compared to parental HeLa cells (Fig. 1g). All these results suggest that resuscitated HeLa cells derived CSCs still kept stable stemness phenotypic characteristics and could be used in the subsequent experiments.

In addition to re-identifying stemness phenotypic characteristics in HeLa cells derived CSCs, we also isolated CSCs derived from SiHa and CaSki cells and identified their stemness phenotypes. As excepted, after approximately 5 weeks of non-adhesive culture, the SFE of SiHa and CaSki cells derived CSCs was obviously higher than that of parental SiHa and CaSki cells (Additional file 1 Figure S1A). In addition, the expression levels of ALDH1, CD49f, Sox2, Nanog, and Oct4 were obviously increased in SiHa and CaSki cells derived CSCs (Additional file 1 Figure S1B). Moreover, we also observed the enhanced migratory ability of SiHa and CaSki cells derived CSCs compared to parental SiHa and CaSki cells along with up-regulated expression of N-cadherin and Vimentin, and down-regulated E-cadherin expression (Additional file 1 Figure S1C-D).

### Zoledronic acid inhibits the proliferation of cervical cancer cells derived CSCs

**Fig. 2:**
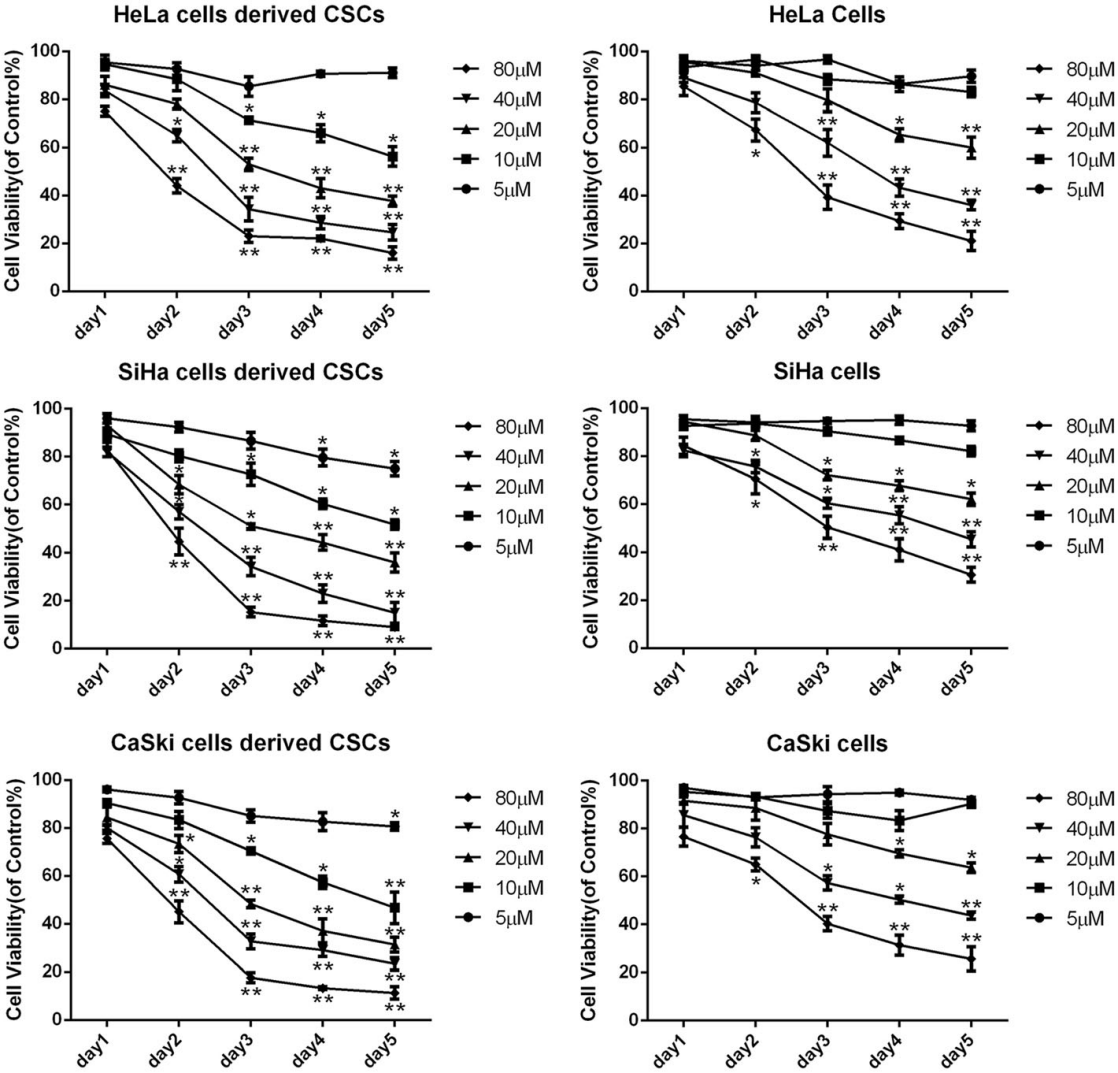
Zoledronic acid inhibits the proliferation of cervical cancer cells derived CSCs in vitro**.** Parental HeLa, SiHa, and CaSki cells as well as their derived CSCs were seeded in 96-well plates and treated with zoledronic acid at different concentrations for 1–5 days. Cell viability was determined by the modified MTT assay. OD values of each treated group were compared with that of the control at the same time point. Control vs. 5, 10, 20, 40, and 80 μM of zoledronic acid: * *P* < 0.05, ** *P* < 0.01. Results are shown as mean values ± SD of independent experiments performed in triplicate

To confirm the cytotoxicity of zoledronic acid on cervical cancer cells derived CSCs or parental cervical cancer cells, we performed the modified MTT assay and the results showed that zoledronic acid inhibited the proliferation of cervical cancer cells derived CSCs as well as their parental cervical cancer cells, as shown in Fig. 2. The IC_50_ at 72 h was 23.39 μM, 21.25 μM, 20.21 μM, 56.26 μM, 64.54 μM, and 55.31 μM in HeLa cells derived CSCs, SiHa cells derived CSCs, CaSki cells derived CSCs, HeLa, SiHa, and CaSki cells, respectively. These results indicate that cervical cancer cells derived CSCs are more responsive to zoledronic acid compared to the parental cervical cancer cells.

### Zoledronic acid weakens the stemness phenotypes of cervical cancer cells derived CSCs

**Fig. 3:**
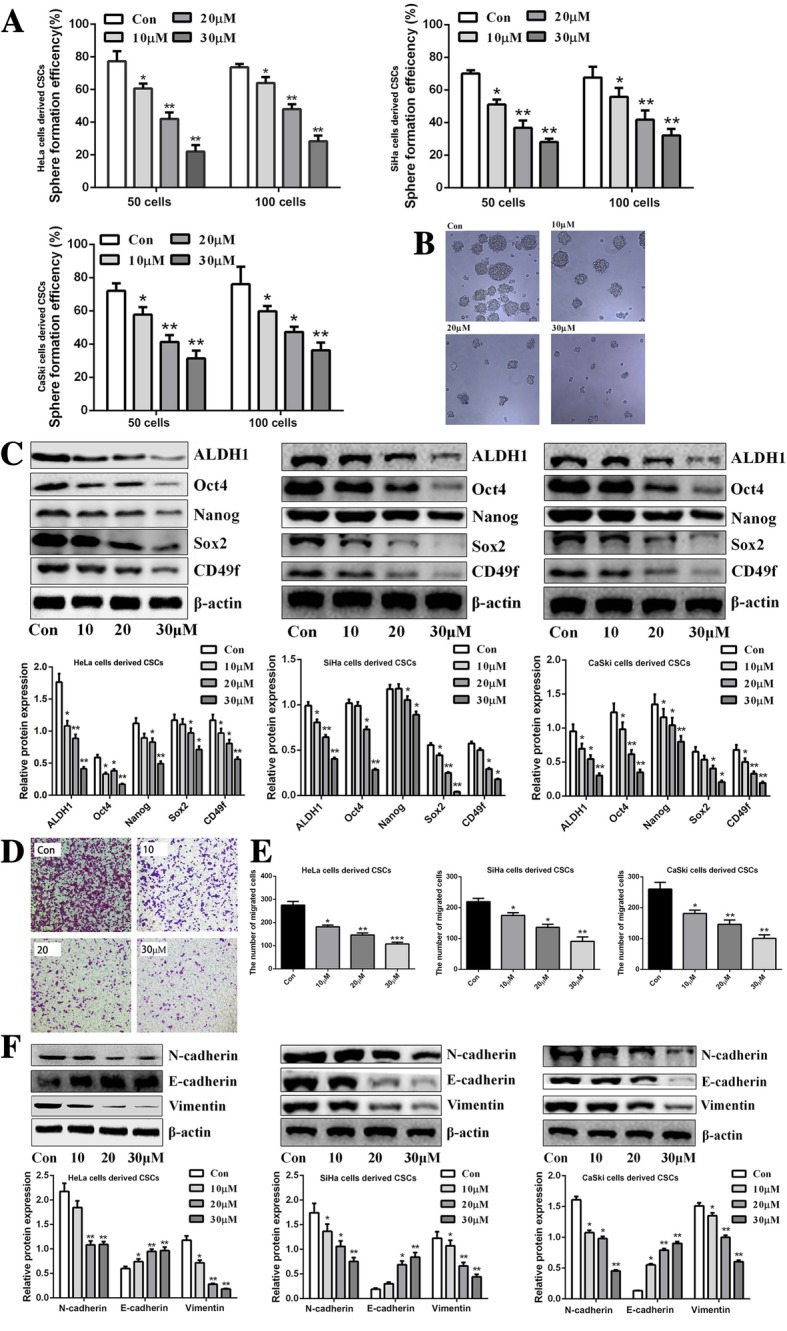
Zoledronic acid weakens the stemness phenotypic characteristics of cervical cancer cells derived CSCs. Approximately 50 and 100 cells derived from cervical cancer cells derived CSCs were seeded in 24-well plates and treated with 10, 20, and 30 μM zoledronic acid. The colonies (> 50 cells) were counted under the microscope. The histograms show the SFE of cervical cancer cells derived CSCs treated with 10, 20, and 30 μM zoledronic acid **(a)**. Representative morphology difference of HeLa cells derived CSCs treated or not with zoledronic acid (10, 20, and 30 μM), original magnification, × 400 **(b)**. Western blot analysis of ALDH1, Sox2, CD49f, Nanog, and Oct4 in cervical cancer cells derived CSCs treated or not with zoledronic acid (10, 20, and 30 μM) **(c)**. The migrated HeLa cells derived CSCs after being treated or not with 10, 20, and 30 μM zoledronic acid, respectively; original magnification, × 200 **(d)**. The histograms show the migrated number of cervical cancer cells derived CSCs **(e)**. Western blot analysis of E-cadherin, N-cadherin, and Vimentin in cervical cancer cells derived CSCs treated or not with zoledronic acid (10, 20, and 30 μM) **(f)**. Control vs. 10, 20, and 30 μM zoledronic acid: * *P* < 0.05, ** *P* < 0.01. Results are shown as mean values ± SD of independent experiments performed in triplicate

Afterwards, the effects of zoledronic acid against cervical cancer cells derived CSCs were explored. As shown in Fig. 3 and Additional file 2 Figure S2A, the SFE of cervical cancer cells derived CSCs was significantly decreased in dose-dependent manners after treatment with 10, 20, and 30 μM zoledronic acid while there was no effects on parental cervical cancer cells. In addition, compared with vehicle, the morphology of cervical cancer cells derived CSCs treated with zoledronic acid were smaller and looser (Fig. 3b). Furthermore, the results showed that the expression levels of ALDH1, Nanog, CD49f, Sox2, and Oct4 were decreased after treatment with zoledronic acid in cervical cancer cells derived CSCs but in parental cervical cancer cells, the expression levels of these stemness markers mentioned above were almost not changed (Fig. 3c and Additional file 2 Figure S2B). Moreover, decreased migratory ability was observed in cervical cancer cells derived CSCs along with down-regulated expression of N-cadherin and Vimentin and up-regulated E-cadherin after treatment with 10, 20, and 30 μM zoledronic acid, respectively (Fig. 3d-f). Nevertheless, in zoledronic acid treated parental cervical cancer cells, the migratory ability as well as the expression of EMT associated markers did not change (Additional file 2 Figure S2C-D).

### Zoledronic acid induces the apoptosis of cervical cancer cells derived CSCs

**Fig. 4:**
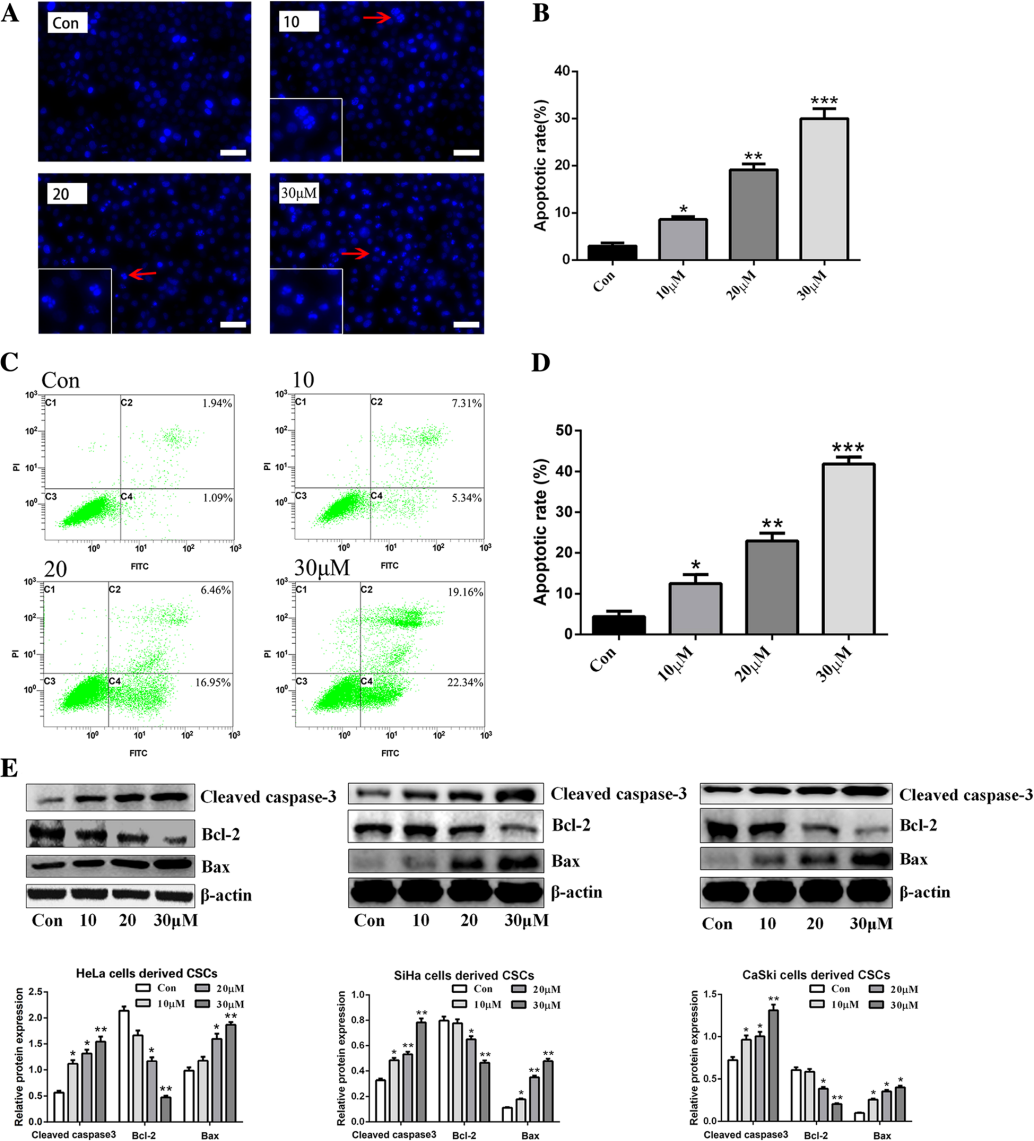
Zoledronic acid induces the apoptosis of cervical cancer cells derived CSCs**.** DAPI staining of HeLa cells derived CSCs after being treated or not with zoledronic acid (10, 20, and 30 μM, respectively). Typical apoptotic bodies in HeLa cells derived CSCs are shown with red arrows **(a)**. The histograms show the proportions of DAPI-stained apoptotic cervical cancer cells derived CSCs **(b)**. Flow cytometry of apoptotic HeLa cells derived CSCs after being treated or not with 10, 20, and 30 μM zoledronic acid; the histogram shows the proportions of apoptotic HeLa cells derived CSCs **(c-d)**. Western blot analysis of Bcl-2, Bax, and Cleaved caspase-3 in cervical cancer cells derived CSCs treated or not with zoledronic acid (10, 20, and 30 μM) **(e)**. Scale bars represent 50 μM in inset. Control vs. 10, 20, and 30 μM of zoledronic acid: * *P* < 0.05, ** *P* < 0.01, *** *P* < 0.001. Results are shown as mean values ± SD of independent experiments performed in triplicate

To confirm the pro-apoptotic effect of zoledronic acid on cervical cancer cells derived CSCs, Annexin V/PI flow cytometry, DAPI staining, and western blot were performed. As shown in Fig. 4a-b, the formation of apoptotic bodies, a typical apoptotic feature, was observed. The apoptotic proportion in untreated HeLa cells derive CSCs was 3.2 ± 0.36% and increased to 8.2 ± 1.26%, 16.8 ± 4.7%, and 36.2 ± 5.21% after treatment with 10, 20, and 30 μM zoledronic acid, respectively. Similarly, the significantly increase of apoptotic bodies in CSCs derived from SiHa or CaSki cells was also verified after zoledronic acid treatment (Additional file 3 Figure S3A). Furthermore, using Annexin V/PI flow cytometry analysis, we verified that zoledronic acid induced the apoptosis of HeLa cells derived CSCs in a dose-dependent manner, with the total apoptotic proportions from 12.6 to 41.5% (Fig. 4c-d). Moreover, western blot revealed that the expression levels of anti-apoptotic Bcl-2 were decreased while pro-apoptotic Bax and Cleaved caspase-3 were increased significantly in cervical cancer cells derived CSCs after zoledronic acid treatment (Fig. 4e). In parental cervical cancer cells, in spite of the treatment with zoledronic acid, there was almost no apoptosis induction (Additional file 3 Figure S3B-C).

### Zoledronic acid induces cell cycle arrest in cervical cancer cells derived CSCs

**Fig. 5:**
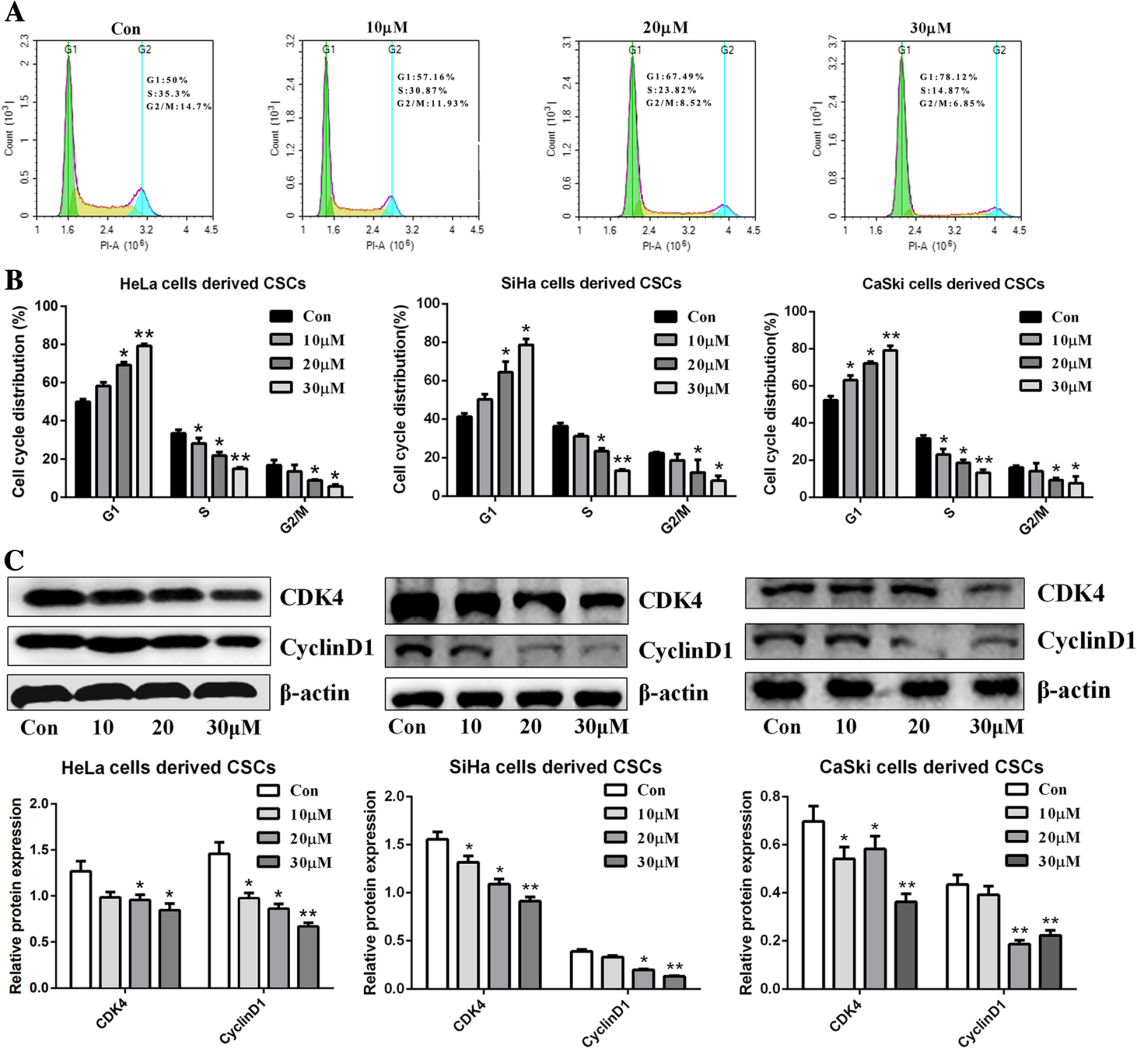
Zoledronic acid promotes cell cycle arrest of cervical cancer cells derived CSCs. Flow cytometry analysis of the cell cycle distribution of HeLa cells derived CSCs treated or not with 10, 20, and 30 μM zoledronic acid, respectively **(a)**. The histograms show the proportions of cell cycle distribution in G1, S, and G2/M phase of cervical cancer cells derived CSCs **(b)**. Western blot analysis of CyclinD1 and CDK4 in cervical cancer cells derived CSCs treated or not with zoledronic acid (10, 20 and 30 μM, respectively) **(c)**. Control vs. 10, 20, and 30 μM of zoledronic acid: * *P* < 0.05, ** *P* < 0.01. Results are mean values ± SD of independent experiments performed in triplicate

To investigate whether zoledronic acid has inhibitory effects on cervical cancer cells derived CSCs, the cell cycle was evaluated. As shown in Fig. 5a-b, zoledronic acid induced significant G1-phase accumulation in dose-dependent manners and the percentage of G1-phase cells was significantly increased. On the other hand, in parental cervical cancer cells, the cell cycle distribution was not disturbed by zoledronic acid (Additional file 4 Figure S4). In addition, western blot further suggested that the expression of CyclinD1 and CDK4 in cervical cancer cells derived CSCs treated with zoledronic acid was decreased in dose-dependent manners (Fig. 5c).

### Anti-cancer efficiency of zoledronic acid against HeLa cells derived CSCs in vivo

**Fig. 6:**
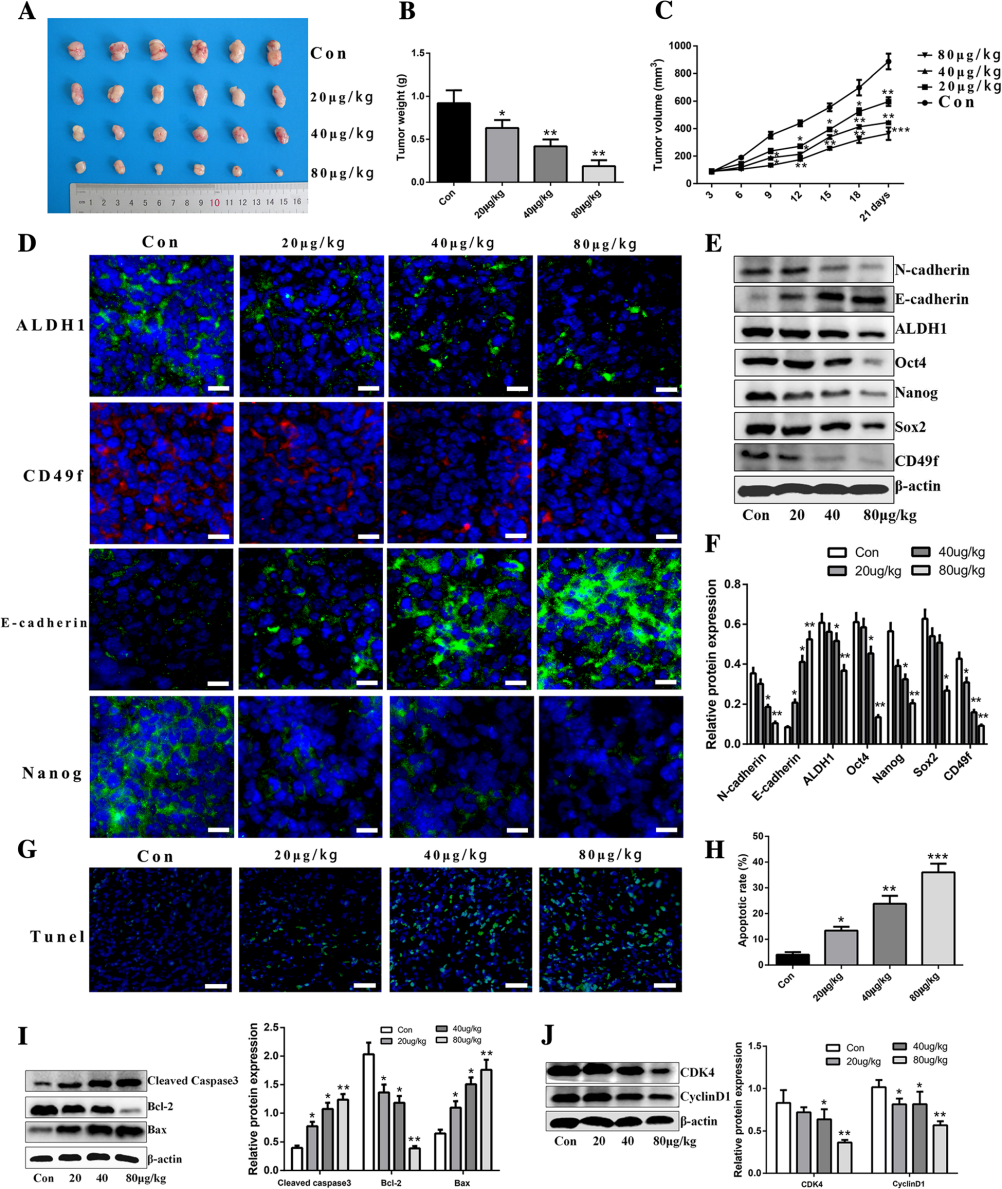
Anti-cancer efficiency of zoledronic acid against HeLa cells derived CSCs in vivo. Tumor tissues derived from HeLa cells derived CSCs bearing nude mice treated with different concentrations of zoledronic acid are exhibited **(a)**. Tumor weights of HeLa cells derived CSCs bearing nude mice administered with different concentrations of zoledronic acid were examined on day 21 **(b)**. Tumor volumes of HeLa cells derived CSCs bearing nude mice were detected every 3 days after treated with different concentrations of zoledronic acid until 21 days **(c)**. Immunofluorescence staining and western blot analysis of stemness- and EMT-associated markers in tumor tissues derived from zoledronic acid-treated HeLa cells derived CSCs bearing nude mice **(d-f)**. TUNEL staining of apoptotic cells in tumor tissues derived from zoledronic acid-treated HeLa cells derived CSCs bearing nude mice **(g)**. The histogram shows the proportions of apoptotic cells **(h)**. Western blot analysis of apoptosis and cell cycle arrest-associated proteins in tumor tissues derived from zoledronic acid-treated HeLa cells derived CSCs bearing nude mice **(i-j)**. Scale bars represent 100 μM or 50 μM in inset. Control vs. 20, 40, and 80 μg/kg of zoledronic acid: * *P* < 0.05, ** *P* < 0.01, *** *P* < 0.001. Results are mean values ± SD of independent experiments performed in triplicate

Next, we investigated whether zoledronic acid could prevent cervical cancer cell derived CSC progression in vivo. As shown in Fig. 6a-c, zoledronic acid suppressed the tumor growth of mice bearing HeLa cells derived CSCs. In addition, the tumor volume and weight in zoledronic acid-treated mice were significantly reduced compared to the control group, especially at doses of 40 and 80 μg/kg. Furthermore, we evaluated whether zoledronic acid suppressed the tumor growth through stemness attenuation, apoptosis induction, and cell cycle arrest in vivo. As expected, immunofluorescence revealed that the expression of ALDH1, Nanog, and CD49f was decreased while E-cadherin expression was increased in zoledronic acid-treated tumor tissues (Fig. 6d). Western blot analysis further confirmed these results (Fig. 6e-f). Moreover, we observed significantly increased apoptotic cells in zoledronic acid-treated tumor tissues using the TUNEL assay (Fig. 6g-h), and the expression of Bcl-2 was up-regulated and Bax was down-regulated (Fig. 6i). Furthermore, the expression of CyclinD1 and CDK4 was significantly lower in tumor tissues from zoledronic acid-treated mice than in tumor tissues from control mice (Fig. 6j).

### Zoledronic acid attenuates the levels of phosphorylated Erk1/2 and Akt in cervical cancer cells derived CSCs

**Fig. 7:**
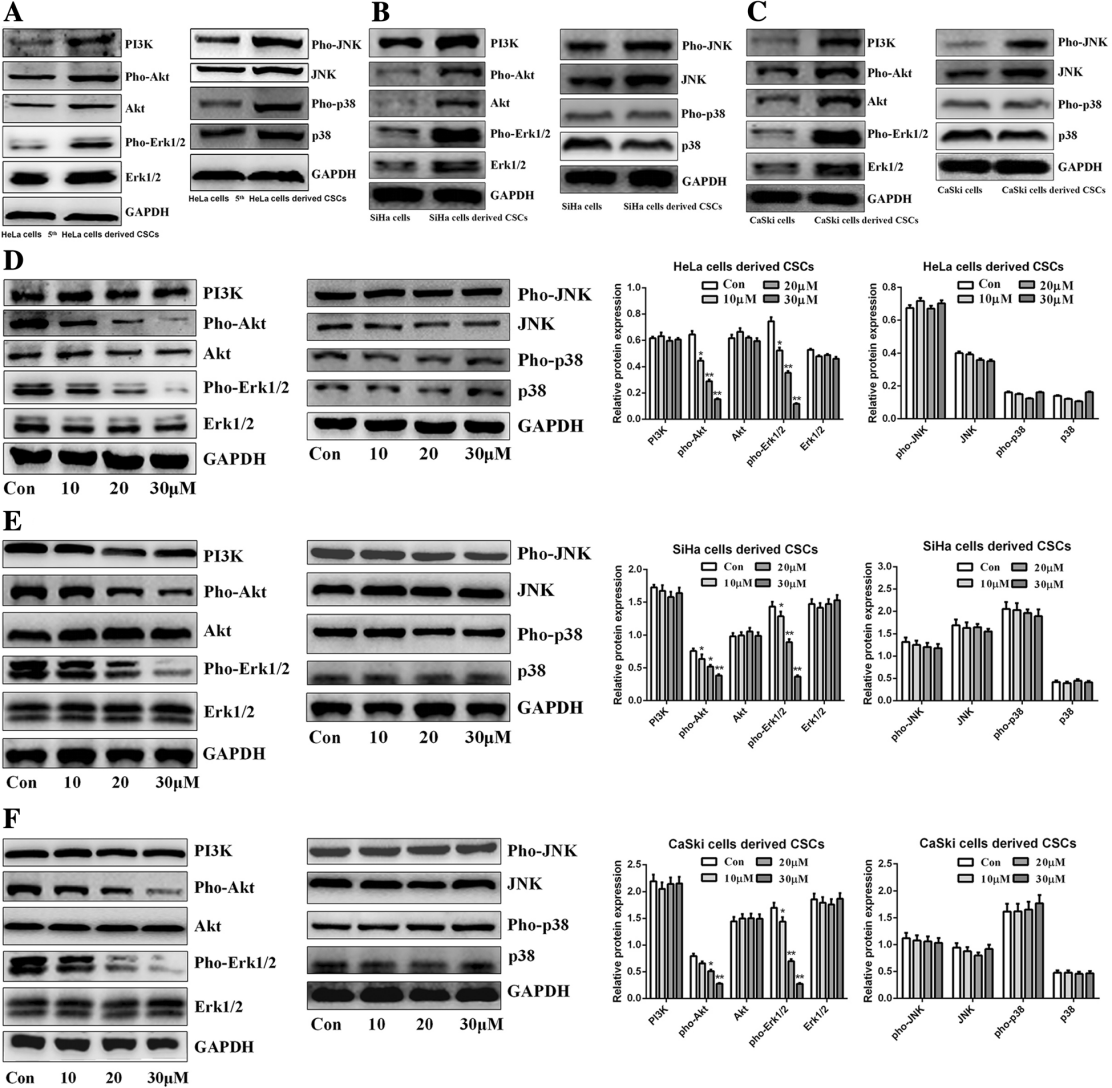
Zoledronic acid attenuates the phosphorylation of Erk1/2 and Akt in cervical cancer cells derived CSCs. Western blot analysis of MAPK- and PI3K/Akt pathways related proteins (total Erk1/2, pho-Erk1/2, pho-JNK, pho-p38, PI3K, total Akt, pho-Akt, total p38, and JNK) in cervical cancer cells derived CSCs and parental cervical cancer cells **(a-c)**. Western blot analysis of total Erk1/2, pho-Erk1/2, pho-JNK, pho-p38, PI3K, total Akt, pho-Akt, total p38, and JNK in cervical cancer cells derived CSCs treated or not with 10, 20, and 30 μM zoledronic acid **(d-f)**. Control vs. 10, 20, and 30 μM of zoledronic acid: * *P* < 0.05, ** *P* < 0.01. Results are shown as mean values ± SD of independent experiments performed in triplicate

For the exploration of the possible molecular mechanisms, we investigated the MAPKs and PI3K/Akt signaling pathways, which are closely associated with CSC maintenance and survival in several types of cancers. We first analyzed the expression of the MAPKs- and PI3K/Akt-related proteins p38, JNK, Erk1/2, PI3K, and Akt, and their phosphorylated forms, between parental HeLa, SiHa, CaSki cells and their derived CSCs. As shown in Fig. 7a, the expression levels of total Erk1/2, pho-Erk1/2, pho-JNK, pho-p38, PI3K, total Akt, and pho-Akt were up-regulated in HeLa cells derived CSCs compared to HeLa cells, but there were no obvious differences regarding total p38 and JNK. In SiHa and CaSki cells as well as their derived CSCs, the expression of total Erk1/2, pho-Erk1/2, total JNK, pho-JNK, PI3K, total Akt, and pho-Akt was up-regulated in SiHa and CaSki cells derived CSCs while there were no significant expression differences of total p38 and pho-p38, compared to parental SiHa and CaSki cells (Fig. 7b-c). Next, we explored the effects of zoledronic acid on the MAPKs and PI3K/Akt pathways in cervical cancer cells derived CSCs. The results showed that the expression of pho-Erk1/2 and pho-Akt was significantly decreased by zoledronic acid, while there was almost no influence on total Erk1/2 and Akt (Fig. 7d-f). We also found that there were no obvious changes in the expression of total p38, pho-p38, total JNK, pho-JNK, and PI3K (Fig. 7d-f). Interestingly, in parental cervical cancer cells, zoledronic acid could not regulate the expression levels of aforementioned MAPK and PI3K/Akt associated proteins (Additional file 5 Figure S5A-C). These results indicate a possible molecular mechanism involved with the suppression of phosphorylated Erk1/2 and Akt in zoledronic acid-treated cervical cancer cells derived CSCs.

### IGF-1 treatment attenuates anti-cancer efficiency of zoledronic acid in HeLa cells derived CSCs

**Fig. 8:**
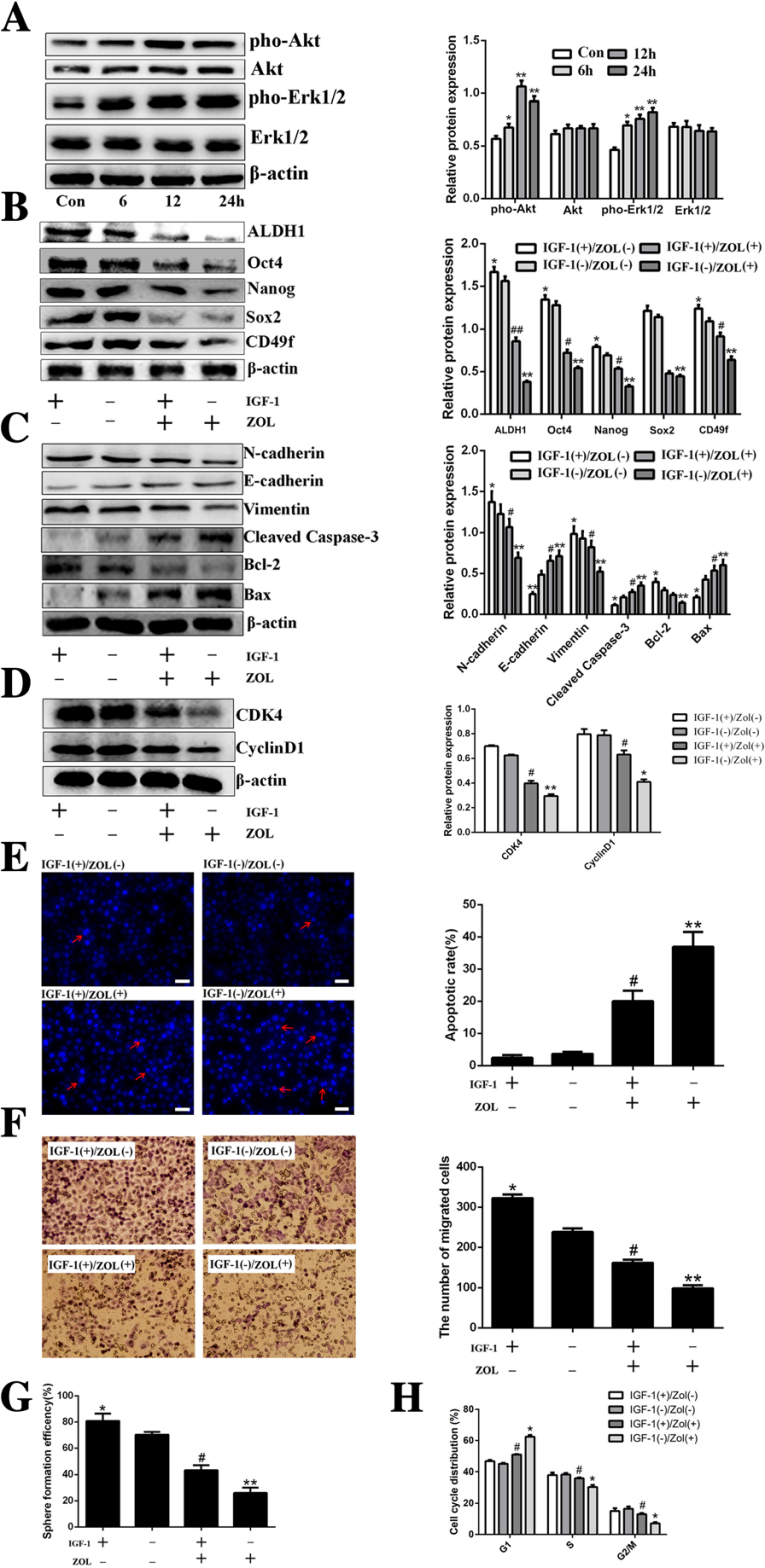
IGF-1 treatment attenuates anti-cancer efficiency of zoledronic acid in HeLa cells derived CSCs. Western blot analysis of phosphorylated Erk1/2 and Akt after treatment with IGF-1 for 6, 12 and 24 h in HeLa cells derived CSCs, respectively **(a)**. Control vs. 6, 12, and 24 h treatment with IGF-1: * *P* < 0.05, ** *P* < 0.01. Western blot analysis of stemness-, EMT-, apoptosis-, and cell cycle-associated proteins in HeLa cells derived CSCs treated or not with IGF-1 and zoledronic acid (ZOL) **(b-d)**. DAPI staining of apoptotic cervical CSCs after being treated or not with IGF-1 and ZOL. Typical apoptotic bodies in HeLa cells derived CSCs are shown with red arrows. The histogram shows the proportions of DAPI-stained apoptotic HeLa cells derived CSCs after being treated or not with IGF-1 and ZOL **(e)**. The migrated HeLa cells derived CSCs after being treated or not with IGF-1 and ZOL. The histogram shows the number of migrated HeLa cells derived CSCs; original magnification, × 200 **(f)**. The histogram shows the SFE of HeLa cells derived CSCs treated or not with IGF-1 and ZOL, respectively (**g**). The histogram shows the proportions of cell cycle distribution in G1, S, and G2/M phases after being treated or not with IGF-1 and ZOL, respectively **(h)**. ZOL(−) plus IGF-1(−) vs. ZOL(+) plus IGF-1(−) or ZOL(−) plus IGF-1(+): * *P* < 0.05, ** *P* < 0.01. ZOL(+) plus IGF-1(−) vs. ZOL(+) plus IGF-1(+): # *P* < 0.05, ## *P* < 0.01. Scale bars represent 50 μM in inset. Results are shown as mean values ± SD of independent experiments performed in triplicate

To further verify the possible involvement of the Erk1/2 and Akt pathways in the stemness phenotype, apoptotic induction, and cell cycle arrest in zoledronic acid-treated cervical cancer cells derived CSCs, IGF-1, a potent stimulator of Erk1/2 and PI3K/Akt pathways [25, 26], was applied. As shown in Fig. 8a, IGF-1 treatment significantly increased the phosphorylated levels of Erk1/2 and Akt in a time-dependent manner, while there was almost no influence on total Erk1/2 and Akt in HeLa cells derived CSCs. Western blot analysis demonstrated that IGF-1 treatment attenuated zoledronic acid triggered changes of Nanog, CD49f, Oct4, ALDH1, Vimentin, N-cadherin, Bcl-2, CyclinD1, and CDK4 (down-regulated) and of E-cadherin, Bax, and Cleaved caspase-3 (up-regulated), while having no significant effect on the expression of Sox2 (Fig. 8b-d). Furthermore, the suppression of the stemness phenotypes including migration and tumor sphere formation, apoptosis induction, and cell cycle arrest evoked by zoledronic acid in HeLa cells derived CSCs were also attenuated after IGF-1 treatment (Fig. 8e-h).

## Discussion

Cervical cancer is the most common gynecologic cancer and the main cause of cancer-related deaths in women all over the world. The major risk factor of cervical cancer is infection by the human papilloma virus (HPV) and other predisposing factors including low socioeconomic background, multiple partners, smoking, early sexual life, and immunosuppression. In spite of screening and anti-cancer therapeutic strategies that improved the prognosis of patients with cervical cancer over the recent years, patients still suffer from recurrence and metastasis, with a 5-year survival rate of < 10%. Therefore, developing new drugs or treatment strategies are important to improve patient prognosis.

CSCs are endowed with stemness phenotypic properties including self-renewal ability, expression of special markers and signal transduction networks, EMT, and chemo/radio-resistance. They are found in various types of tumors and cancer cell lines, including cervical cancer [27]. Recently, increasing scientific evidence indicated that CSCs present in tumors or hematological neoplasms might be closely involved in cancer recurrence or metastasis and are responsible for the failure of anti-cancer therapies [28, 29]. Therefore, developing new drugs or exploring new mechanisms of current drugs targeting CSCs seems to be a promising anti-cancer therapeutic strategy and urgently needed.

Self-renewal ability, including tumor sphere formation in vitro or tumorigenesis in vivo, is the major characteristic of CSCs [30–32]. In the present study, through continuous passages of resuscitated HeLa cells derived CSCs, we could observe increasing and then near stable SFE from the 1^st^- to the 5^th^-passaged HeLa cells derived CSCs. CD49f, Nanog, and ALDH1 are closely related to the stemness phenotype of cervical CSCs [27, 33, 34]. In the present study, we revealed that resuscitated HeLa cells derived CSCs displayed high expression levels of CD49f, Nanog, ALDH1, Sox2, and Oct4 compared to the parental HeLa cells. In addition, tumorigenesis of the 5^th^-passaged HeLa cells derived CSCs indicated that the tumorigenic ability of these cells was significantly higher than in the parental HeLa cells. EMT plays a critical role in the generation and maintenance of CSCs [35, 36]. Our present study indeed observed increased migratory ability and the up-regulated expression of EMT associated markers (N-cadherin and Vimentin) in HeLa cells derived CSCs compared to parental HeLa cells. All these findings suggest that the HeLa cells derived CSCs used in this study harbor stemness in spite of the previous report indicating that lung CSCs suffering from attenuation of stemness-associated markers when experiencing cryopreservation and resuscitation [37]. Moreover, in the present study, we also identified and enriched CSCs derived from other cervical cancer cell lines SiHa and CaSki. These results indicated that, similar with HeLa cells derived CSCs, the stemness phenotypic characteristics including enhanced SFE, overexpressed stemness associated markers and EMT also exited in SiHa and CaSki cells derived CSCs.

Zoledronic acid is widely used to prevent osteolysis and skeletal-related events in advanced cancer patients who develop or suffer from bone metastasis. Recently, emerging evidence indicated that zoledronic acid might be a multiple targeted drug with effects on several types of cancer cells as well as on the tumor microenvironment [16, 38–40]. In this study, the results revealed that cervical cancer cells derived CSCs were more responsive to zoledronic acid compared to the parental HeLa cells or other cervical cancer cells. Overexpression of the ATP-binding cassette (ABC) transporter proteins and evasion of apoptosis existed in CSCs might be the primary reasons for the resistance to chemotherapeutic agents [41, 42]. Nevertheless, researchers also found that some drugs targeting special markers or signaling pathways of CSCs could be more efficient at killing CSCs than standard cancer cells [43, 44]. Indeed, zoledronic acid attenuated the stemness phenotypic characteristics of cervical cancer cells derived CSCs including tumor sphere formation, down-regulating expression levels of stemness- or EMT-associated markers, as well as migratory ability but no effects on parental cervical cancer cells. Previous studies indicated that zoledronic acid induces apoptosis in various type of cancer cells such as lung cancer [45] and myeloma [46]. In this study, we revealed that zoledronic acid significantly induced the apoptosis of cervical cancer cells derived CSCs in dose-dependent manners while we could not observe apoptosis induction of zoledronic acid in parental cervical cancer cells. Previous studies indicated that cell cycle arrest in the G1 phase is mainly mediated through the decreased expression of the cell cycle-associated proteins CyclinD1 and CDK4 [47, 48]. Therefore, cell cycle was examined and the results showed that zoledronic acid induced cycle arrest of cervical cancer cells derived CSCs in the G1 phase. The alteration of CyclinD1 and CDK4 validated these results. However, in parental cervical cancer cells, through flow cytometry analysis, cell cycle distribution was not disturbed by zoledronic acid. We speculated that this could be because zoledronic acid acts on the stemness features of the CSCs, upon which CSCs rely for survival, on pathways required for CSC survival, or on survival pathways that are more activated in CSCs than in cancer cells.

Taken together, all the results suggest that zoledronic acid attenuates the stemness phenotype of cervical cancer cells derived CSCs, arrests these cells in G1 phase, and increases apoptosis, leading to decreased tumor formation both in vitro and in vivo.

To help elucidate the possible molecular mechanisms of zoledronic acid attenuating the stemness phenotype, inducing cell cycle arrest and apoptosis of cervical cancer cells derived CSCs, as suggested above, we investigated two important molecular signaling pathways. The MAPKs and PI3K/Akt pathways are both activated by a number of extracellular signals and growth factors including IGF-1, EGFR, CXCL12, and Six1, and regulate fundamental cellular processes such as proliferation, apoptosis, and cell cycle arrest [49, 50]. Importantly, recent studies indicated that the MAPKs and PI3K/Akt pathways play important functional roles in some types of CSCs derived from colorectal and liver cancers [51, 52]. A review suggested that the PI3K/Akt/mTOR signaling pathway could be an appropriate target for CSC targeted drugs [53]. In prostate CSCs, zoledronic acid increased apoptosis and blocked cell progression through BCL2 and caspases [54]. Nevertheless, whether zoledronic acid regulates the stemness phenotype, cell cycle arrest, and apoptosis of cervical CSCs through the MAPKs and PI3K/Akt signaling pathways remains unclear. In the present study, the results showed that, compared to parental cervical cancer cells, the expression levels of PI3K, total Erk1/2, and Akt were up-regulated in cervical cancer cells derived CSCs; moreover, the levels of phosphorylated Erk1/2 and Akt in these cells were also significantly increased. Previous report indicated that PI3K/Akt/mTOR signaling pathway in breast CSCs is activated and quercetin, a natural product extracted from *Sophora flavescens* Ait, targets this pathway to influence the stemness phenotype of CSCs [12]. The study by Li et al. [12]. supports the concept that sensitive CSCs should be targeted in order to prevent tumor growth, recurrence, and metastasis. Next, we verified that zoledronic acid significantly decreased the phosphorylation of Erk1/2 and Akt, but had almost no effects on the expression of total Ekr1/2 and Akt as well as on PI3K, JNK, p38, pho-JNK, and pho-p38 in cervical cancer cells derived CSCs. Interestingly, in parental cervical cancer cells, the expression of MAPKs- and PI3K/Akt-related proteins we analyzed above showed almost no changes in spite of zoledronic acid treatment. These results suggest that zoledronic acid targeted cervical cancer cells derived CSCs possibly by suppressing phosphorylated Erk1/2 and Akt and this might be closely associated with the sensitivity of zoledronic acid on cervical cancer cells derived CSCs but not the parental cervical cancer cells.

**Fig. 9:**
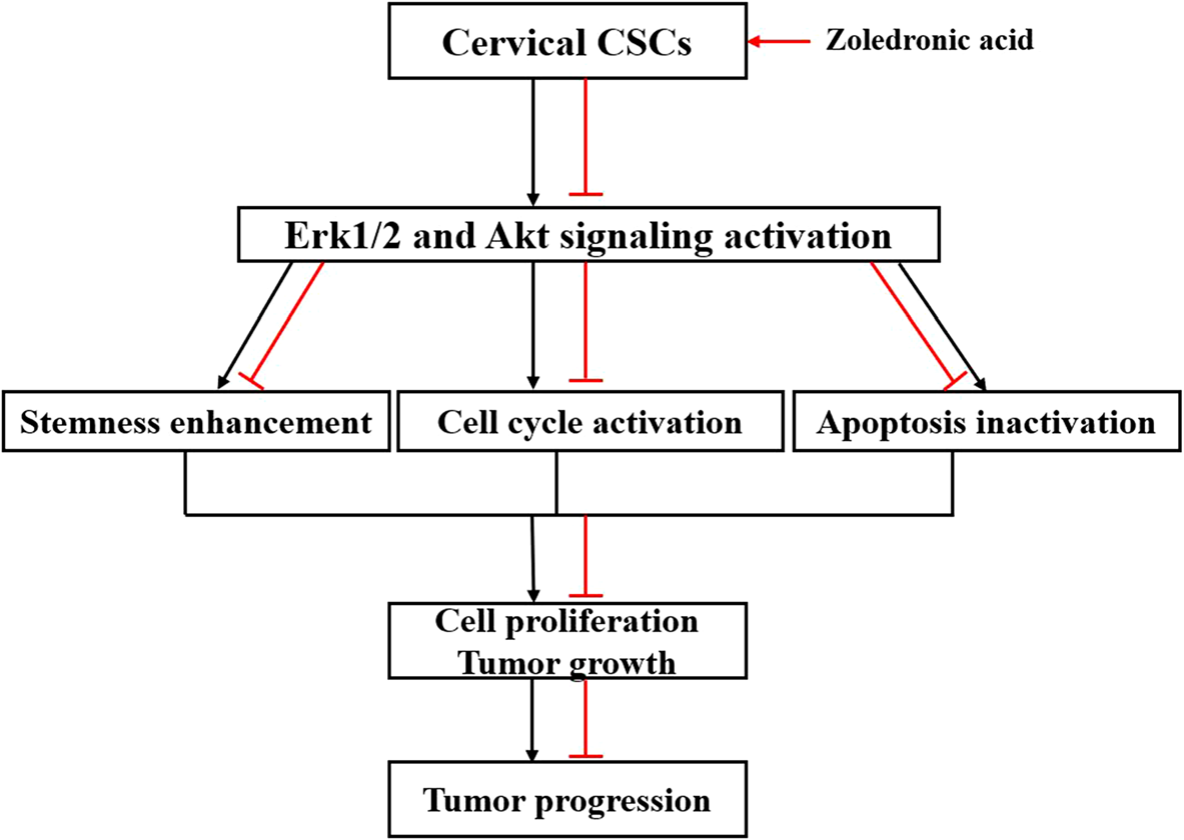
Schematic representation for the outcome of this study

IGF-1 is a potent stimulator of the Erk1/2 and PI3K/Akt pathways [25, 26]. IGF-1 is involved in promoting the mitogenic, metastatic, and antiapoptotic features of many cancer cells, contributing to the maintenance of cancer cells and progression of cancer [55]. In order to demonstrate that the effects of zoledronic acid involved the regulation of the Erk1/2 and PI3K/Akt pathways, IGF-1 was added to observe the changes in stemness phenotype, apoptosis, and cell cycle after zoledronic acid treatment. The results indicated that IGF-1 attenuated the anti-cancer efficiency of zoledronic acid on HeLa cells derived CSCs, strongly suggesting that the effects of zoledronic acid on cervical CSCs are mediated, at least in part, by the Erk1/2 and PI3K/Akt pathways. Figure 9 provides a schematic representation of the outcome of this study.

## Conclusions

Taken together, the present study suggests that zoledronic acid inhibits the growth of cervical cancer cells derived CSCs through stemness attenuation, apoptosis induction, and cell cycle arrest. The possible molecular mechanisms might be closely involved with, at least in part, the suppression of phosphorylated Erk1/2 and Akt. Therefore, zoledronic acid might be a novel targeted drug against cervical CSCs and could provide a new and promising strategy for anti-cancer therapy and deserves to be explored in further.

## Additional files

Additional file 1: **Figure S1.** Identification of the stemness phenotypic characteristics of SiHa and CaSki cells derived CSCs**.** The graphs show the SFE of SiHa and CaSki cells derived CSCs as well as parental SiHa and CaSki cells **(a)**. Western blot analysis of ALDH1, Sox2, CD49f, Nanog, and Oct4 in SiHa and CaSki cells derived CSCs as well as parental SiHa and CaSki cells **(b)**. The histograms show the number of migrated SiHa and CaSki cells derived CSCs as well as parental SiHa and CaSki cells **(c)**. Western blot analysis of E-cadherin, Vimentin, and N-cadherin in SiHa and CaSki cells derived CSCs as well as parental SiHa and CaSki cells **(d)**. * *P* < 0.05, ** *P* < 0.01, *** *P* < 0.001. Results are shown as mean values ± SD of independent experiments performed in triplicate. (TIF 1052 kb)

Additional file 2: **Figure S2.** Zoledronic acid affects the cervical cancer cells with stemness phenotype. Approximately 50 and 100 cells derived from cervical cancer cells were seeded in 24-well plates and treated with 10, 20, and 30 μM zoledronic acid. The colonies (> 50 cells) were counted under the microscope. The histograms show the SFE of HeLa, SiHa, and CaSki cells treated with 10, 20, and 30 μM zoledronic acid **(a)**. Western blot analysis of ALDH1, Sox2, CD49f, Nanog, and Oct4 in HeLa, SiHa, and CaSki cells treated or not with zoledronic acid (10, 20, and 30 μM) **(b)**. The histograms show the migrated number of HeLa, SiHa, and CaSki cells treated with 10, 20, and 30 μM zoledronic acid **(c)**. Western blot analysis of E-cadherin, Vimentin, and N-cadherin in HeLa, SiHa, and CaSki cells treated or not with zoledronic acid (10, 20, and 30 μM) **(d)**. Control vs. 10, 20, and 30 μM zoledronic acid: * *P* < 0.05, ** *P* < 0.01. Results are shown as mean values ± SD of independent experiments performed in triplicate. (TIF 1756 kb)

Additional file 3: **Figure S3.** Zoledronic acid induces apoptosis of cervical cancer cells. The histograms show the proportions of DAPI-stained apoptotic SiHa cells derived CSCs, CaSki cells derived CSCs as well as HeLa, SiHa, and CaSki cells after being treated or not with zoledronic acid (10, 20, and 30 μM) **(a-b)**. Western blot analysis of Bcl-2 and Bax in HeLa, SiHa, and CaSki cells treated or not with zoledronic acid (10, 20, and 30 μM) **(c)**. Control vs. 10, 20, and 30 μM zoledronic acid: * *P* < 0.05, ** *P* < 0.01. Results are shown as mean values ± SD of independent experiments performed in triplicate. (TIF 819 kb)

Additional file 4: **Figure S4.** Zoledronic acid arrests the cell cycle of cervical cancer cells. The histograms show the proportions of cell cycle distribution in G1, S, and G2/M phase of HeLa, SiHa, and CaSki cells through flow cytometry analysis. Control vs. 10, 20, and 30 μM of Zoledronic acid: * *P* < 0.05, ** *P* < 0.01. Results are shown as mean values ± SD of independent experiments performed in triplicate. (TIF 380 kb)

Additional file 5: **Figure S5.** Zoledronic acid acts through the MPAK- and PI3K/Akt-pathways related proteins of cervical cancer cells. Western blot analysis of total Erk1/2, pho-Erk1/2, pho-JNK, pho-p38, PI3K, total Akt, pho-Akt, total p38, and JNK in HeLa, SiHa, and CaSki cells treated or not with 10, 20, and 30 μM zoledronic acid **(a-c)**. Control vs. 10, 20, and 30 μM of Zoledronic acid: * *P* < 0.05, ** *P* < 0.01. Results are shown as mean values ± SD of independent experiments performed in triplicate. (TIF 1378 kb)

## Abbreviations

CSCs: Cancer stem cells
EGFR: Epidermal growth factor receptor
EMT: Epithelial mesenchymal transition
SFE: Sphere formation efficiency
MAPK: Mitogen activated protein Kinase
PBS: Phosphate Buffer Saline
FBS: Fetal bovine serum
DMEM: Dulbecco’s modified Eagle’s medium
HPV: Human papillomavirus
