# Supplementary figures and images for: Zoledronic acid inhibits the growth of cancer stem cell derived from cervical cancer cell by attenuating their stemness phenotype and inducing apoptosis and cell cycle arrest through the Erk1/2 and Akt pathways
Source: J Exp Clin Cancer Res. 2019 Feb 21;38:93. doi: 10.1186/s13046-019-1109-z (PMC6385443; doi:10.1186/s13046-019-1109-z)

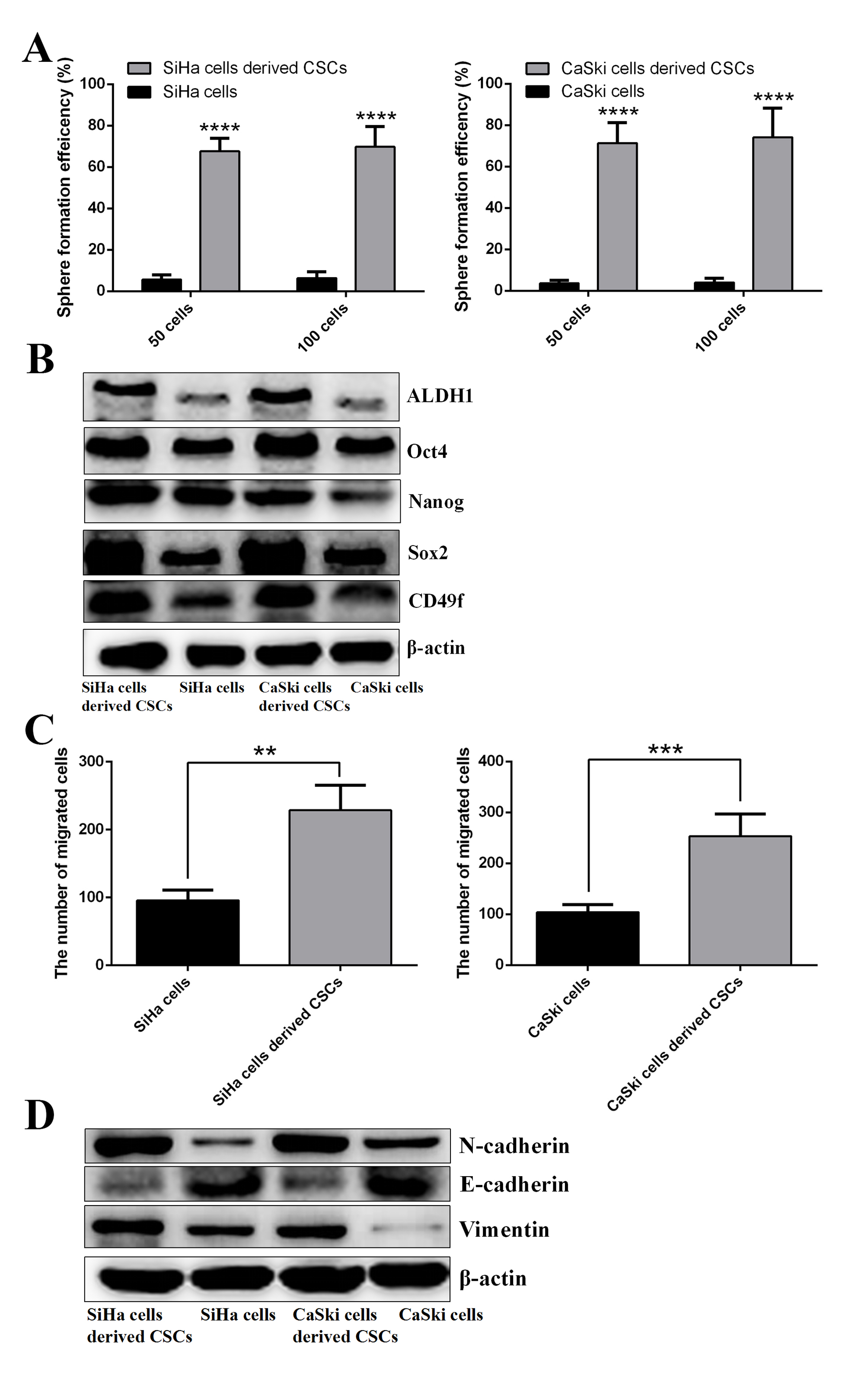

Supplement: Supplementary file 1 — Figure S1. Identification of the stemness phenotypic characteristics of SiHa and CaSki cells derived CSCs. The graphs show the SFE of SiHa and CaSki cells derived CSCs as well as parental SiHa and CaSki cells (a). Western blot analysis of ALDH1, Sox2, CD49f, Nanog, and Oct4 in SiHa and CaSki cells derived CSCs as well as parental SiHa and CaSki cells (b). The histograms show the number of migrated SiHa and CaSki cells derived CSCs as well as parental SiHa and CaSki cells (c). Western blot analysis of E-cadherin, Vimentin, and N-cadherin in SiHa and CaSki cells derived CSCs as well as parental SiHa and CaSki cells (d). * P < 0.05, ** P < 0.01, *** P < 0.001. Results are shown as mean values ± SD of independent experiments performed in triplicate. (TIF 1052 kb) [file 13046_2019_1109_MOESM1_ESM.tif]

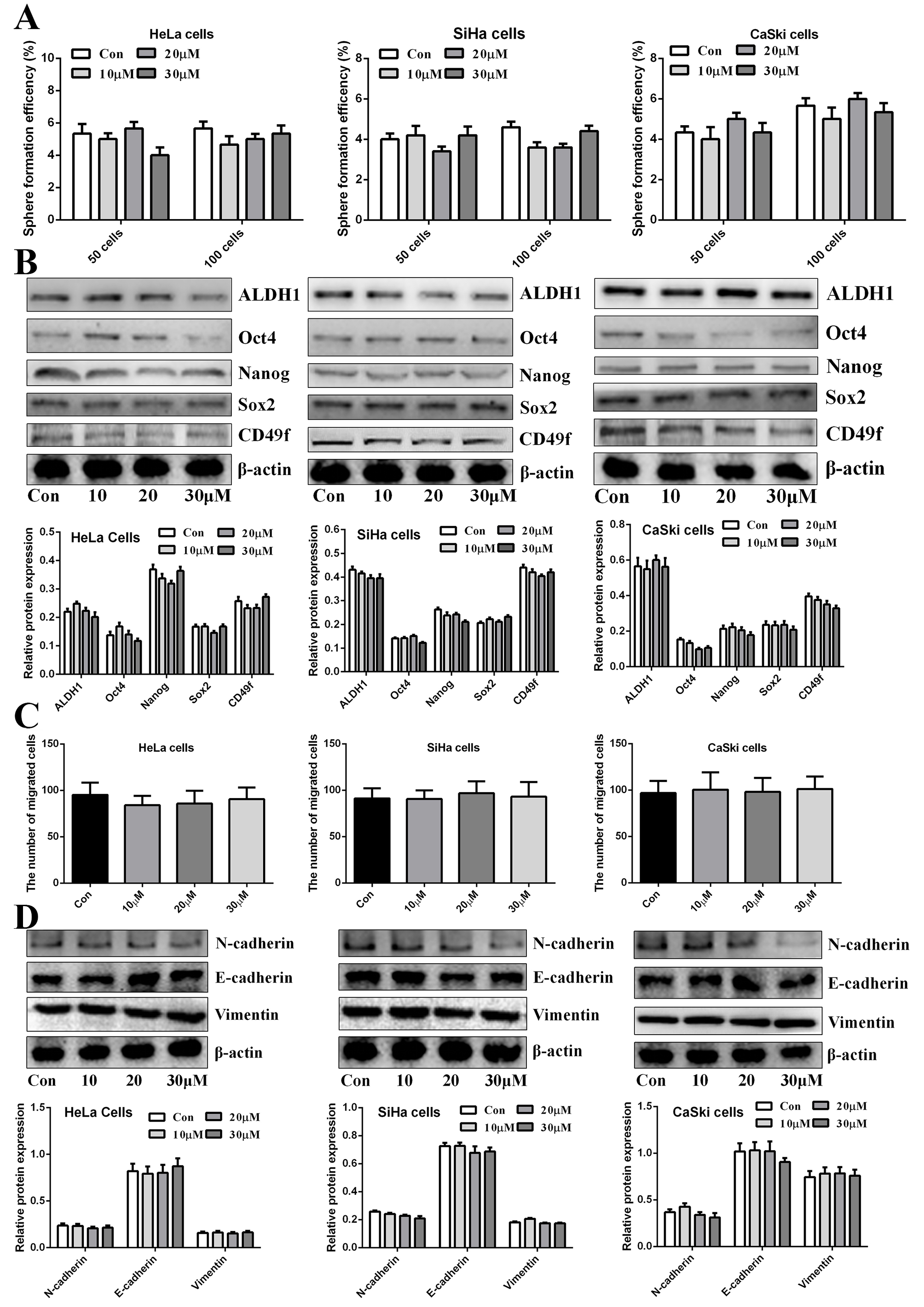

Supplement: Supplementary file 2 — Figure S2. Zoledronic acid affects the cervical cancer cells with stemness phenotype. Approximately 50 and 100 cells derived from cervical cancer cells were seeded in 24-well plates and treated with 10, 20, and 30 μM zoledronic acid. The colonies (> 50 cells) were counted under the microscope. The histograms show the SFE of HeLa, SiHa, and CaSki cells treated with 10, 20, and 30 μM zoledronic acid (a). Western blot analysis of ALDH1, Sox2, CD49f, Nanog, and Oct4 in HeLa, SiHa, and CaSki cells treated or not with zoledronic acid (10, 20, and 30 μM) (b). The histograms show the migrated number of HeLa, SiHa, and CaSki cells treated with 10, 20, and 30 μM zoledronic acid (c). Western blot analysis of E-cadherin, Vimentin, and N-cadherin in HeLa, SiHa, and CaSki cells treated or not with zoledronic acid (10, 20, and 30 μM) (d). Control vs. 10, 20, and 30 μM zoledronic acid: * P < 0.05, ** P < 0.01. Results are shown as mean values ± SD of independent experiments performed in triplicate. (TIF 1756 kb) [file 13046_2019_1109_MOESM2_ESM.tif]

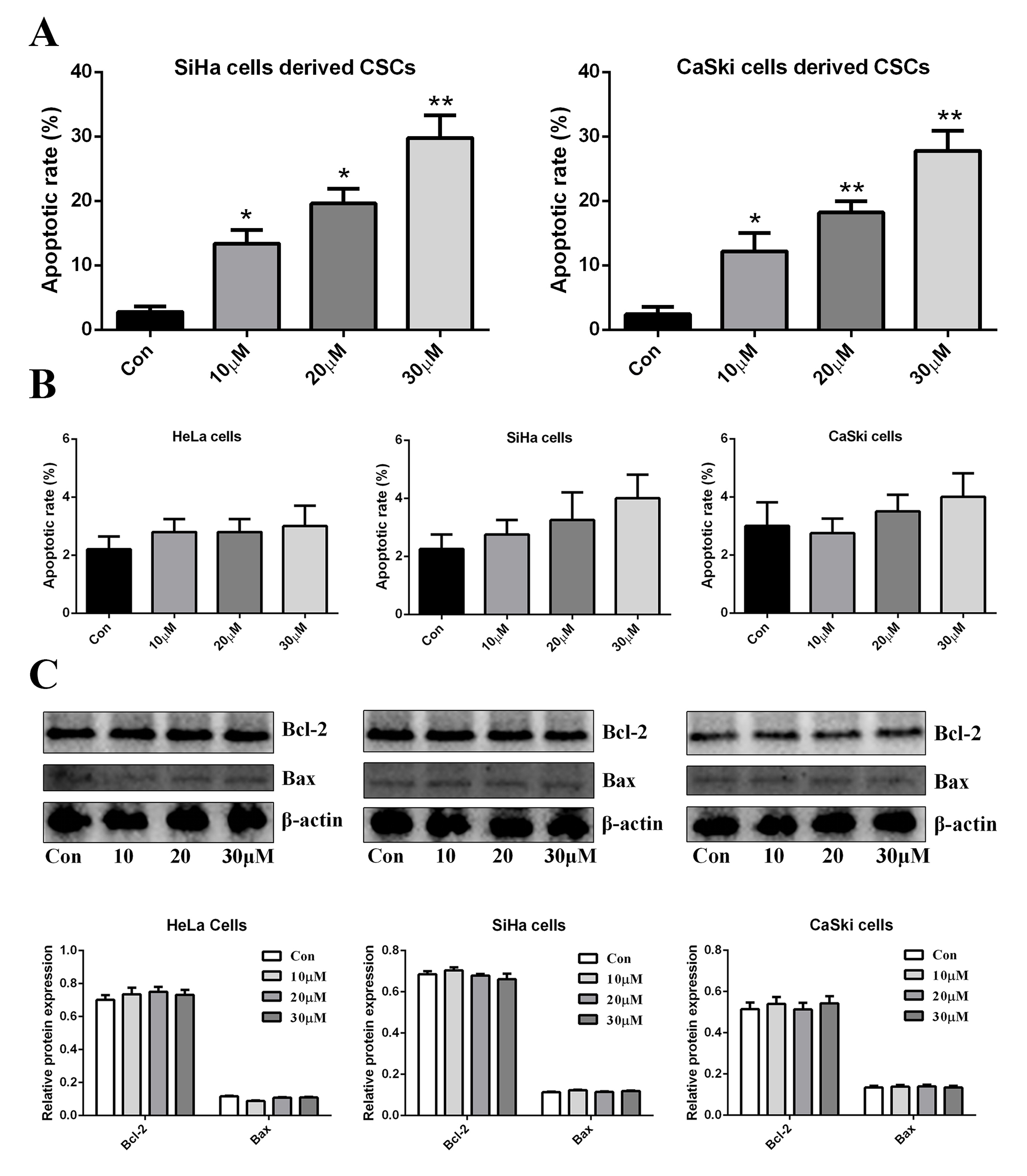

Supplement: Supplementary file 3 — Figure S3. Zoledronic acid induces apoptosis of cervical cancer cells. The histograms show the proportions of DAPI-stained apoptotic SiHa cells derived CSCs, CaSki cells derived CSCs as well as HeLa, SiHa, and CaSki cells after being treated or not with zoledronic acid (10, 20, and 30 μM) (a-b). Western blot analysis of Bcl-2 and Bax in HeLa, SiHa, and CaSki cells treated or not with zoledronic acid (10, 20, and 30 μM) (c). Control vs. 10, 20, and 30 μM zoledronic acid: * P < 0.05, ** P < 0.01. Results are shown as mean values ± SD of independent experiments performed in triplicate. (TIF 819 kb) [file 13046_2019_1109_MOESM3_ESM.tif]

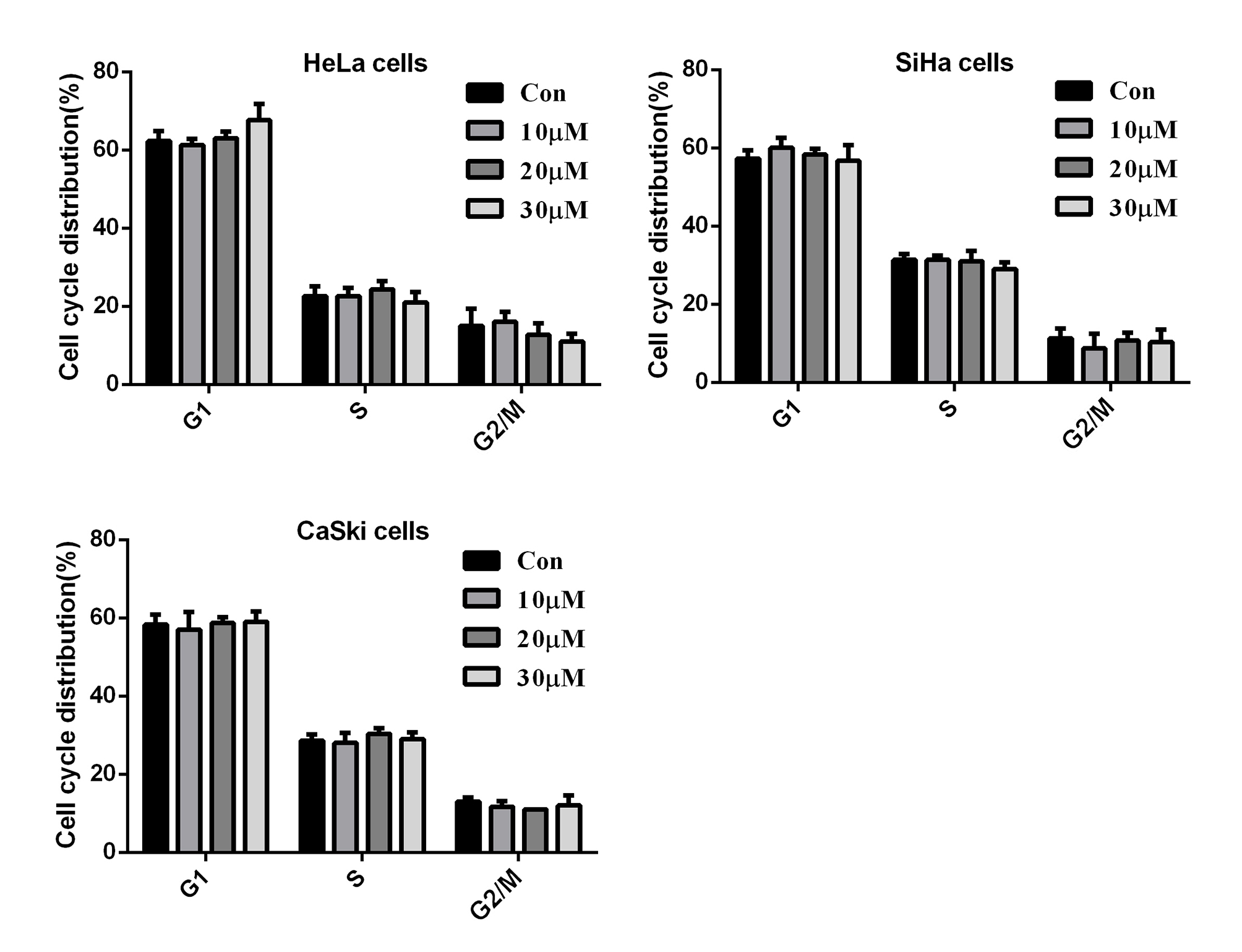

Supplement: Supplementary file 4 — Figure S4. Zoledronic acid arrests the cell cycle of cervical cancer cells. The histograms show the proportions of cell cycle distribution in G1, S, and G2/M phase of HeLa, SiHa, and CaSki cells through flow cytometry analysis. Control vs. 10, 20, and 30 μM of Zoledronic acid: * P < 0.05, ** P < 0.01. Results are shown as mean values ± SD of independent experiments performed in triplicate. (TIF 380 kb) [file 13046_2019_1109_MOESM4_ESM.tif]

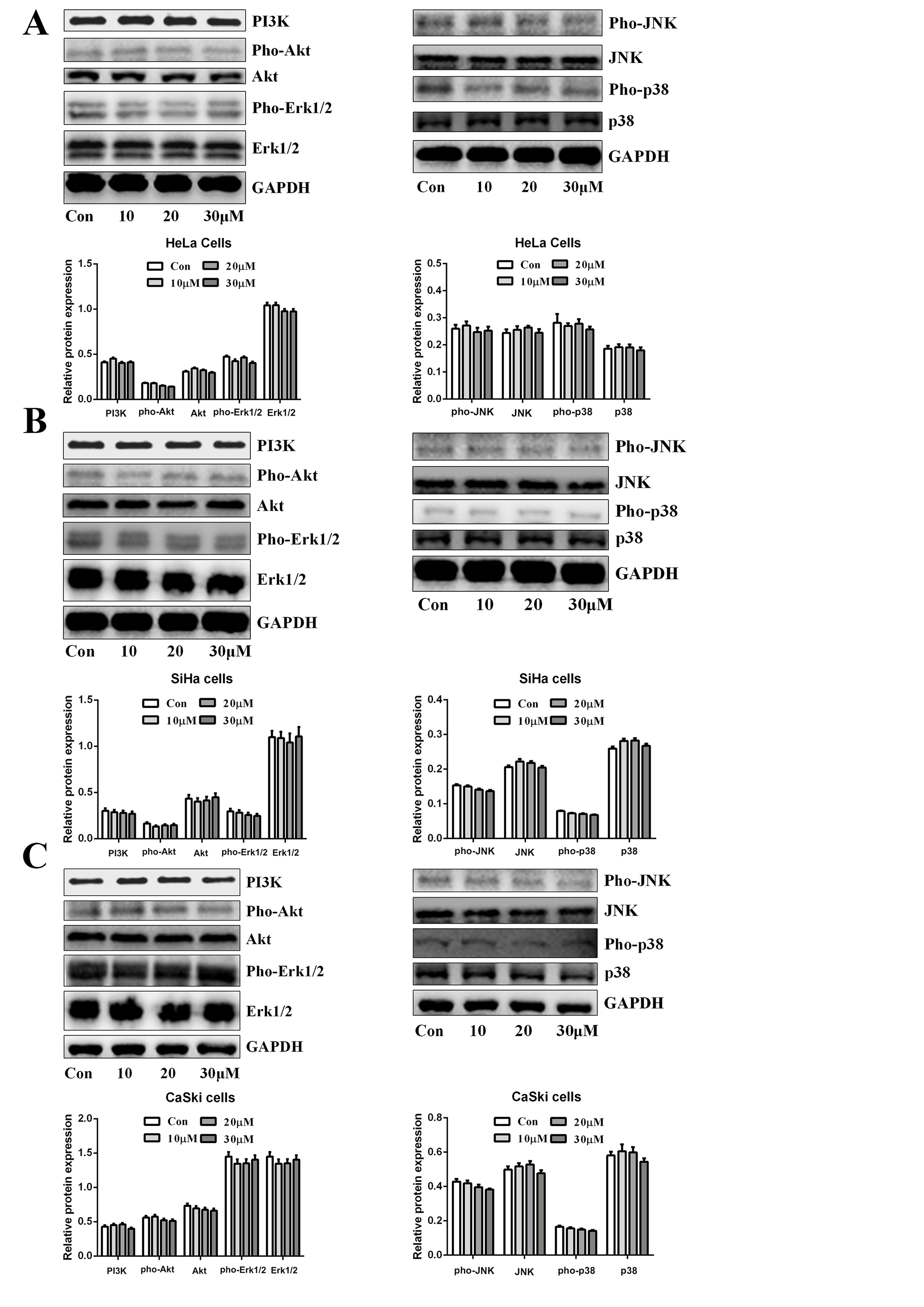

Supplement: Supplementary file 5 — Figure S5. Zoledronic acid acts through the MPAK- and PI3K/Akt-pathways related proteins of cervical cancer cells. Western blot analysis of total Erk1/2, pho-Erk1/2, pho-JNK, pho-p38, PI3K, total Akt, pho-Akt, total p38, and JNK in HeLa, SiHa, and CaSki cells treated or not with 10, 20, and 30 μM zoledronic acid (a-c). Control vs. 10, 20, and 30 μM of Zoledronic acid: * P < 0.05, ** P < 0.01. Results are shown as mean values ± SD of independent experiments performed in triplicate. (TIF 1378 kb) [file 13046_2019_1109_MOESM5_ESM.tif]
